# A “proto” type galectin expressed in striped bass (*Morone saxatilis*) tissues is released to epidermal mucus and binds to bacterial and mucus glycans

**DOI:** 10.3389/fcimb.2025.1572734

**Published:** 2025-05-14

**Authors:** Davin E. Henrikson, Hafiz Ahmed, Satoshi Tasumi, Mahesh Gokara, Chiguang Feng, Kelsey Abernathy, Muddassar Iqbal, Mario A. Bianchet, Gerardo R. Vasta

**Affiliations:** ^1^ Department of Microbiology and Immunology, University of Maryland School of Medicine, University of Maryland Baltimore (UMB), Baltimore, MD, United States; ^2^ Institute of Marine and Environmental Technology, Columbus Center, Baltimore, MD, United States; ^3^ Department of Immunology and Cancer Biology, Mayo Clinic, Jacksonville, FL, United States; ^4^ Department of Biophysics and Biophysical Chemistry, Johns Hopkins University School of Medicine, Baltimore, MD, United States

**Keywords:** galectin, teleost fish, macrophages, epidermal mucus, bacterial recognition

## Abstract

Like all aquatic vertebrates and invertebrates, teleost fish are subject to the constant pressure of bacterial, fungal, and parasitic organisms present in the environmental interface that can potentially cause disease. Numerous defense molecules, including galectins, have been isolated from the skin and gut tissues of several marine and freshwater fish species. To provide new insights into the potential role(s) of galectins in the teleost fish innate immune system, we carried out studies on the striped bass (*Morone saxatilis*), a keystone fish species in Chesapeake Bay. We purified from epidermal skin mucus, and skin and muscle tissue, a 15-kDa galectin that we designated Msgal1-L1 (*M. saxatilis* galectin1-like protein 1). Both the transcript sequence and gene organization of Msgal1-L1 suggested a close relationship to the zebrafish galectin Drgal1-L2 and other proto type galectins from vertebrates, including the mammalian galectin-1. Glycan microarray analysis of Msgal1-L1 revealed a binding preference for Galβ1,4GlcNAc, and a homology structural model identified the amino acids involved in ligand recognition, both observations consistent with proto type galectins. Immunohistological examination localized Msgal1-L1 to epithelial and macrophage-/fibroblast-like cells in mucosal tissues, including skin and gill. The preliminary localization of Msgal1-L1 in free macrophage-like cells in epidermal mucus was corroborated by immunofluorescence analysis of macrophages isolated from head kidney. Msgal1-L1 binds in a carbohydrate-specific manner to O-glycosylated components of epidermal mucus. Msgal1-L1 agglutinated environmental bacterial species and strains, some of which are recognized fish pathogens, such as *Vibrio* and *Edwardsiella* spp. A microbial microarray analysis revealed that it preferentially binds to bacterial exopolysaccharides (e.g., *Streptococcus* and *Shigella* spp.) as well as various lipopolysaccharide O-antigen serotypes of *Proteus* spp. A preliminary solid-phase assay showed that Msgal1-L1 strongly bound *Streptococcus* sp., but very weakly to *Mycobacterium marinum*, an endemic pathogen of striped bass in Chesapeake Bay. Taken together, this evidence suggests that Msgal1-L1 may function in defense recognition against environmental bacteria by agglutinating and/or cross-linking them to mucus oligosaccharides to immobilize them within the epidermal mucus film and prevent their access to the fish epithelial cell surface. *M. marinum* would evade this defense mechanism to reach and infect the fish skin epithelial layer.

## Introduction

In addition to the constant pressure on marine, estuarine, and freshwater natural fish populations caused by overfishing and detrimental environmental changes, fish stocks must contend with the environmental infectious challenge of viruses, bacteria, and fungi, some of which may be members of the normal microbiota (Harvell et al., 1999; Okon et al., 2024). In this regard, the interface of the fish with their environment, which includes epithelial surfaces of skin, gills, and gut that have been shown as routes for microbial infection, also displays abundant mucus-producing cells (goblet, club, and sacciform cells) that secrete a continuous mucus film that coats these epithelial surfaces (Bansil and Turner, 2017; Dash et al., 2018; Reverter et al., 2018). The epidermal mucus layer functions not only as a lubricant for the mucosal epithelia but also as the first line of defense against environmental pathogens, constituting a viscous mechanical barrier that it is regularly sloughed off and prevents pathogens from colonizing on the epithelial surface (Boutin et al., 2013; Gomez et al., 2013; Benhamed et al., 2014; Reverter et al., 2018; Seo et al., 2020). Most importantly, fish mucus contains innate immune factors (e.g., lectins, antimicrobial peptides, lysozyme, and complement) that can actively immobilize and kill the potential pathogens (Tasumi et al., 2002; Subramanian et al., 2007; Jurado et al., 2015; Brinchmann, 2016; Guardiola et al., 2016; Sanahuja et al., 2019).

Among the numerous defense molecules that have been isolated from fish mucus, lectins, sugar-binding proteins, have attracted considerable attention due to their binding properties for glycans present on the surface of potential pathogens, including viruses, bacteria, and fungi, as well as eukaryotic parasites (Suzuki et al., 2003; Vasta et al., 2011). Animal lectins have been classified into over a dozen families based on their requirement of divalent cation requirements for ligand binding, the presence of conserved sequence motifs in their carbohydrate binding domains (CRDs), and, most recently, their structural folds (Vasta and Ahmed, 2008). Lectin families of wide taxonomic distribution include the C-, F-, I-, and P-type lectins, ficolins, and galectins (Vasta and Ahmed, 2008). Among these, the galectins, formerly known as S-type lectins, are a functionally diverse family of β-galactoside-binding lectins that are present in virtually all animal species, from invertebrates to vertebrates, including man (Di Lella et al., 2011; Cummings et al., 2022). Based on the structural organization of their peptide subunits, galectins are classified into “proto,” “chimera,” and “tandem-repeat” types (Hirabayashi and Kasai, 1993) and designated by numbers, following the order of their discovery (Di Lella et al., 2011; Cummings et al., 2022). In vertebrates, the proto-type galectin-1 (Gal-1), the chimera-type galectin-3 (Gal-3), and the tandem-repeat galectin-9 (Gal-9) are the best characterized examples (Liao et al., 1994; Di Lella et al., 2011; Cummings et al., 2022). Proto- and chimera-type galectins display a single CRD per peptide subunit, while in the tandem-repeat-type galectins, two CRDs are joined by a flexible linker peptide (Hirabayashi and Kasai, 1993). Galectin subunits can form multivalent oligomeric structures, such as dimers (e.g., Gal-1) or pentamers (e.g., Gal-3) (Di Lella et al., 2011; Cummings et al., 2022).

Since their discovery in the early 1980s, galectins have been identified as lectins that bind to endogenous (“self”) glycans and mediate key functions such as early development and tissue repair (Barondes et al., 1994; Colnot et al., 1997; Craig et al., 2010; Vasta et al., 2017; de Jong et al., 2020), angiogenesis (Croci et al., 2014), pregnancy (Blois et al., 2007, 2019), and regulation of immune homeostasis (Rabinovich and Croci, 2012). Furthermore, galectins have been recently shown to play critical roles in pathological processes in man: cancer, obesity, inflammation, and diabetes (Rabinovich et al., 2007; Liu et al., 2012; Rabinovich and Conejo-Garcia, 2016; Menéndez-Huergo et al., 2017). During the last few years, however, mounting experimental evidence has revealed that galectins of all three major types can also bind to exogenous (“non-self”) glycans on the surface of potentially pathogenic viruses, bacteria, yeasts, and eukaryotic parasites, and function as pattern recognition receptors and effector factors in innate immune responses of both invertebrate and vertebrate species (Vasta, 2009; Vasta et al., 2012, 2017; Baum et al., 2014). Although several articles have reported the presence of galectins in the skin/epidermal mucus of teleost fish (Shiomi et al., 1989; Muramoto et al., 1999; Tasumi et al., 2004; Tsutsui et al., 2019; Takayama et al., 2009; Nakamura et al., 2007), relevant aspects of the structural, biochemical, and functional aspects of the mucus galectins, as well as the tissues or cells that may be source(s) of these proteins have not been fully elucidated.

The striped bass (*Morone saxatilis*, Walbaum 1792) also known as rockfish, is a teleost fish species of high environmental and economic importance along the Atlantic coast of the USA, and particularly in Chesapeake Bay. It is the largest member of the family Percichthydae (order Perciformes) and is an anadromous, euryhaline species, usually found in rivers and bays from the St. Lawrence River to northern and western Florida (northern Gulf of Mexico) to Louisiana (Harding and Mann, 2003). This fish species has been an important resource along the Atlantic coast of the United States since colonial times, and it continues to be one of the most sought-after commercial and recreational finfish in the Chesapeake Bay and constitutes the basis of a widely distributed and highly relevant and economically successful aquaculture industry for the Mid-Atlantic region (Engle and Senten, 2023; Kenter and Berlinsky, 2023). Both natural and farmed striped bass populations, however, are susceptible to bacterial infections (Plumb, 1991; Jacobs et al., 2009). Wild caught animals are frequently infected with *Mycobacterium marinum* and related mycobacterial species, and display skin lesions, granulomas, and ulcers (Overton et al., 2003; Rhodes et al., 2004; Gauthier and Rhodes, 2009; Delghandi et al., 2020). Transmission of *Mycobacterium* spp. to humans can take place by handling of infected animals (Hashish et al., 2018), and outbreaks have been documented in zebrafish research facilities (Mason et al., 2016). Although *M. marinum* infections in fish populations are considered endemic to the Chesapeake Bay, the high susceptibility of striped bass remains to be fully understood.

In this report, we describe the biochemical, genomic, and structural characterization of a 15-kDa galectin (Msgal1-L1) present in epidermal tissues and muscle, macrophages, and mucus of striped bass, and characterize its binding properties for mucus glycans and environmental bacteria, some of them well-recognized fish pathogens, which suggest a defensive role of this galectin at the fish epidermal mucus/environmental interphase.

## Experimental procedures

### Animals and collection of epidermal mucus and selected tissues

Healthy adult striped bass (*M. saxatilis*) weighing between approximately 3 and 5 pounds were obtained from the in-house Aquaculture Research Center at the Institute of Marine and Environmental Technology. Animals were anesthetized with 25–100 mg/L Tricaine (MS-222) buffered with equal weight of NaHCO_3_. Epidermal mucus was immediately collected by gently scraping with a metal spatula the skin in a head-to-tail direction and kept on wet ice until processing. This was followed by euthanasia by either severing the spinal cord posterior to the gill slits or by overdose of phenoxyethanol (0.079 ppt). Selected tissues and organs were immediately dissected from animals and portions from each were processed for protein purification, immunohistology analysis, or isolation of head kidney macrophages, or frozen in liquid nitrogen, and stored at −80°C until further processing for nucleic acid extraction.

### Purification of a galectin from striped bass tissues

A galectin was isolated and purified from striped bass muscle using an improved protocol as previously reported (Ahmed et al., 1996). Briefly, dissected muscle tissue was homogenized in ME/[PBS/10]/Lac [1:10 phosphate buffered saline (0.01 M Na_2_HPO_4_/0.15 M NaCl/0.01% NaN_3_, pH 7.5) containing 10 mM β-mercaptoethanol (ME) and 0.1 M lactose] supplemented with 1 mM phenylmethylsulfonyl fluoride (PMSF) and clarified by centrifugation. The clear supernatant was passed through DEAE-Sepharose pre-equilibrated with ME/[PBS/10], eluted with 5 bed volumes of ME/PBS/0.5 M NaCl, and absorbed on a lactosyl-Sepharose column pre-equilibrated with ME/PBS/0.5 M NaCl. After the column was washed with ME/PBS/0.5 M NaCl and equilibrated with ME/[PBS/10], the galectin was eluted with at least 2 bed volumes of ME/[PBS/10]/0.1 M lactose. The protein containing fractions were absorbed on a DEAE-Sepharose column (1 mL bed volume) in the presence of 1:1 ME/[PBS/10]:glycerol and stored at −20°C until use. Similar procedures were used for striped bass skin and epidermal mucus.

### Analytical procedures

Agglutination tests with human erythrocytes (Referencells, Immucor, Norcos, GA) were carried out in BSA (bovine serum albumin)-coated 96 well Terasaki plates (Robbins Scientific, Mountain View, CA) as reported earlier (Vasta and Marchalonis, 1986). Protein concentrations were determined with the Bio-Rad Protein Assay using BSA as standard (Vasta and Marchalonis, 1986). Analytical polyacrylamide gel electrophoresis (PAGE) in the presence of sodium dodecyl sulfate (SDS; 2%) was carried out on 15% (w/v) acrylamide gels under reducing conditions as reported elsewhere (Vasta et al., 1986). For SDS-PAGE on tricine gels, glycine was replaced by tricine. The molecular weight of the native protein was estimated by gel permeation chromatography carried out on a Pharmacia Superose 6 column as previously described (Vasta and Marchalonis, 1986; Ahmed et al., 1996).

Analytical isoelectric focusing (IEF) of the Msgal1-L1 was carried out on a thin (1 mm) layer of 5% polyacrylamide Ampholine PAGplate (Pharmacia, pH range 3–10) as previously described (Ahmed et al., 1996). For two-dimensional (2D) electrophoresis, IEF was first performed using the Protean IEF System with immobilized pH gradient (IPG) strips (Bio-Rad) as follows: 100 μg of Msgal1-L1 was loaded onto rehydrated IPG strip, pH 3–10, and focused for 30,000 V/h. Strips were equilibrated in dithiothreitol (DTT) equilibration buffer (2% w/v DTT, 6 M urea, 2% SDS, 20% glycerol, and 0.05 M Tris-HCl, pH 8.8) followed by iodoacetamide equilibration buffer (2.5% w/v iodoacetamide, 6 M urea, 2% SDS, 20% glycerol, and 0.05 M Tris-HCl, pH 8.8) and then transferred to a precast gel (Criterion, Bio-Rad) for second dimension. Separated proteins were transferred to PVDF and detected by Ponceau Red staining followed by Western blot (WB) using standard protocols.

### Preparation of rabbit anti-Msgal1-L1 antiserum

Anti-Msgal1-L1 antiserum was prepared in New Zealand white rabbits at Duncroft (Lovetsville, FL) by multiple subcutaneous and intramuscular injections of affinity-purified Msgal1-L1 (100 μg/injection), and the antibody titer was determined by enzyme-linked immunosorbent assay (ELISA) as previously described (Ahmed et al., 1996). The specificity of the antiserum was assessed by WB by comparing its binding to the purified Msgal1-L1 and the crude skin and muscle extracts as previously described (Ahmed et al., 2004).

### Amino acid sequencing of Msgal1-L1

Automated Edman degradation of affinity-purified Msgal1-L1 (Hewick et al., 1981) was performed at the Winship Cancer Center’s Microchemical Facility, Emory University School of Medicine (Atlanta, GA) as a service. Sample preparation, fractionation of peptides, and sequencing of the fractionated peptides were carried out following the protocols previously described (Ahmed et al., 1996).

### Cloning and sequencing of an Msgal1-L1 transcript

Total RNA was isolated from the selected tissues using the RNeasy kit (Qiagen) and poly (A)+ RNA was isolated from the total RNA on poly (dT)-Dynabeads using an mRNA purification kit (Dynal, Oslo, Norway). The first-strand cDNA was synthesized using the First-strand cDNA Synthesis kit (Life Sciences). Using the peptide sequence data obtained for Msgal1-L1, several degenerate primers were designed and used to PCR amplify the first-strand cDNA. All PCR amplifications were carried out with Taq DNA polymerase (Promega) according to the manufacturer’s instructions, and cloning and sequencing were performed as previously described (Ahmed et al., 2004). All other manipulations of nucleic acids such as ligation, transformation, gel electrophoresis, gel elution, and preparation of buffers were carried out following standard protocols (Sambrook et al., 1989). Amplicons generated from the initial PCR amplification were sequenced and gene-specific primers (**Table 1**) were designed to obtain the full-length cDNA. For this purpose, the 5′ and 3′ Rapid Amplification of cDNA Ends (RACE) of Msgal1-L1 were performed with gene-specific primers using the Marathon cDNA Amplification Kit (Clontech) according to the manufacturer’s protocol and the full-length cDNA was reconstructed from the overlapping RACE products.

**Table 1:** Primers for Msgal1-L1 gene amplification.

Target	FORWARD (5'>3')	REVERSE (5'>3')
full length	GCACATATGTTTAATGGTTTGCTCATA	GGTGGATCCTTATTTGATCTCAAGG
Upstream	GTAATACGACTCACTATAGGGC (AP1)	GCAAACCATTAAACATGATTGCAGATG
Intron I	TCTCACTTCTCCTCAGCTGTACTTGAC	ATGGTCTGCCCGACCTTGAAGGAC
Intron II	TTCA AGGTCGGGCAGACCATGACC	GACCACCACATTCTCG TCTCC
Intron III	GAGGCTTTCCTTTCCAACAGGG	ATCCCCAACAAAGTTGATGAAGGAGTAC
Downstream	GAGGCTTTCCTTTCCAACAGGG	GTAATACGACTCACTATAGGGC (AP1)
cDNA for LIC	GACGACGACAAGATGTTTAATGG TTGC TC	GAGGAGAAGCCCGGTTCCTTAT TTGATC TC
3' RACE-1 -2	ACCAGACGCCTCGCGGCACTCCC TGGTGTGAGGAGCACCGTGAGGGAGG	
5' RACE-1 -2	ATCCCCAACAAAGTTGATGAAGGAGTAC AAGTTGCAGGTTTATTGATCTCA	

### Isolation and characterization of the Msgal1-L1 gene

For extraction of DNA, the blood collected by tail venipuncture was mixed with PBS/EDTA and placed on wet ice to prevent clotting and DNA degradation. High-molecular-weight genomic DNA was extracted using the GenomicPrep Kit (Amersham Biosciences, Piscataway, New Jersey) according to the manufacturer’s instructions. For Southern blot, genomic DNA was digested with *Bam*HI*, Xba*I, and *Ssp*I, gel-electrophoresed, denatured, neutralized following standard protocols (Sambrook et al., 1989), and transferred to a Nytran SuPerCharge Nylon membrane (Schleicher & Schuell, Keene, New Hampshire) using the TurboBlotter System (Schleicher & Schuell) following the manufacturer’s protocol. DNA was covalently cross-linked to the damp membrane using a UV Stratalinker 2400 (Stratagene, La Jolla, California) by exposing blot to a total dose of 120 mJ/cm^2^ and the detection was performed using a DIG-labeled probe and reagents from the DIG High Prime DNA Labeling and Detection Starter Kit II (Roche Applied Science, Indianapolis, Indiana).

To determine the Msgal1-L1 gene organization, the “Gene Walker” DNA Walking Kit (Clontech) was used to amplify and sequence unknown regions of *Msgal1-L1*. For this purpose, five genomic libraries were prepared using restriction enzymes *Pvu*II, *Sca*I, *Eco*RV, *Stu*I, and *Dra*I, ligated with the Genome Walker Adapter (Clontech) according to the manufacturer’s protocols. With gene-specific primers (**Table 1**) and adapter-specific primers (AP-1), suppression PCR was performed to amplify the 5′ upstream region, 3′ downstream region, and introns of Msgal1-L1 gene.

### Phylogenetic analysis of Msgal1-L1

Phylogenetic analysis of Msgal1-L1 was performed using the ClustalW program, using the NJ (Neighbor Joining) method of Saitou and Nei (1987), and included representative prototype galectins from mammals, birds, and tunicates. Analysis was also carried out with Msgal1-L1 and fish galectin genomic and cDNA sequences available in GenBank and genome databases of TIGR and HGMP Resource Centre (Fugu Genomics Group) to investigate evolutionary relationships and structural conservation.

### Homology model of the Msgal1-L1 structure

#### Template search with BLAST and HHBlits (HMM-HMM-based lightning-fast iterative sequence search)

The target sequence was searched with BLAST against the primary amino acid sequences contained in the SWISS-MODEL template library (SMTL). A total of 190 templates were found. An initial HHblits profile was built using the procedure outlined in Remmert et al. (2012) followed by one iteration of HHblits against NR20. The obtained profile was then searched against all profiles of the SMTL, yielding 316 templates for consideration.

#### Construction of the Msgal1-L1 model

Models were built based on the target-template alignment using ProMod3, a homology modeling tool in the SWISS-MODEL pipeline. Conserved coordinates between the target and template were copied directly to the model, while insertions and deletions were modeled using a fragment library. Side chains were reconstructed, and the model’s geometry was optimized using a force field. Additionally, an alternative model was generated with PROMOD-II (Guex et al., 2009; Remmert et al., 2012).

#### Model quality estimation

The global and per-residue model quality was assessed using the QMEAN scoring function (Benkert et al., 2010). For improved performance, weights of the individual QMEAN terms were trained specifically for SWISS-MODEL.

### Expression and purification of recombinant Msgal1-L1

A full-length cDNA coding for Msgal1-L1 was subcloned from pGEM-T cloning vector (Promega) into the pET-30 bacterial expression vector (Novagen) using *Nde*I and *BamH*I restriction sites and transformed into *Escherichia coli* strain BL21 (DE3) competent cells. After induction with 1 mM IPTG, the cells were harvested, lysed, and the recombinant Msgal1-L1 (rMsgal1-L1) was purified on a lactose-Sepharose affinity column. The purified protein was approximately 19.5 kDa, consistent with the predicted size of Msgal1-L1 with an N-terminal S- and His-tag. Following cleavage of the tags with enterokinase, rMsgal1-L1 displayed the same electrophoretic mobility as the native Msgal1-L1 in a 15% SDS-PAGE gel (**Supplementary Figure 1**). The agglutinating activity of rMsgal1-L1 was compared to the native Msgal1-L1 using the protocol described in the previous section. In some experiments, rMsgal1-L1 was biotinylated as previously described (Nita-Lazar et al., 2015).

### Carbohydrate specificity

#### Preparation of the rMsgal1-L1–HRP conjugate

The rMsgal1-L1 was first carboxamido-methylated with iodoacetamide on a DEAE-Sepharose column (0.5 mL bed volume) in the presence of lactose as previously described (Ahmed et al., 1996). After washing the column with [PBS/10], the carboxamido-methylated (CAM)-rMsgal1-L1 was eluted with 0.5 M NaCl/PBS pH 7.4 and conjugated to horseradish peroxidase (HRP) through glutaraldehyde coupling (Ahmed et al., 1996). Briefly, the conjugation mixture was diluted 40-fold with cold water and adsorbed onto DEAE-Sepharose (0.5 mL) preequilibrated with [PBS/10]. After the column was washed, the conjugate was eluted with 2 mL of l.0 M NaCl and PBS, pH 7.5, and purified on a lactosyl-Sepharose column. Finally, the conjugate was separated from the unreacted rMsgal1-L1 by gel permeation chromatography on a Superose 6 column as described above. The purified rMsgal1-L1–HRP conjugate was stored in 1% BSA and 50% glycerol at −20°C.

#### Solid-phase binding-inhibition assay

Binding of the rMsgal1-L1–HRP to asialofetuin (ASF) and its inhibition by carbohydrates were assessed and optimized as reported elsewhere (Ahmed et al., 1996, 2002). Fetuin is a highly sialylated glycoprotein that, when enzymatically or chemically desialylated (ASF), displays the abundant subterminal galactosyl moieties to which most galectins avidly bind. Thus, the inhibition of galectin binding to immobilized ASF by mono- and oligosaccharides is a standard system to analyze the fine specificity of novel galectins. Briefly, ASF (0.5 μg/100 μl/well) in 0.1 M Na_2_CO_3_/0.02% NaN_3_ (pH 9.6) was adsorbed onto the plastic wells (37°C, 3 h) and the bound glycoprotein was fixed with 2% formaldehyde (37°C, 30 min). The plates were washed three times with PBS (azide-free)/0.05% Tween 20, and incubated with the Msgal1-L1–HRP conjugate (10 ng/100 μl/well for binding assays) or with a pre-incubated mixture of equal volume of conjugate and varying concentrations of test ligands (for binding-inhibition assays). After incubation (4°C, 1 h) the plates were washed with ice-cold azide-free PBS-Tween 20 buffer and the bound peroxidase activity was assayed with 2,2’-azino-bis(3-ethylbenzothiazoline-6-sulfonic acid) (ABTS) as previously described (Ahmed et al., 1996).

***Glycan microarray analysis***. Analysis of the carbohydrate specificity of Msgal1-L1 was carried out at the National Center for Functional Glycomics (NCFG) at Beth Israel Deaconess Medical Center, Harvard Medical School, on version 5.0 of the CFG Glycan Microarray printed with 562 glycans in replicates of six. The His-tagged rMsgal1-L1 (1 mg/mL) was dialyzed against PBS containing 10 mM mercaptoethanol (PBS/2-ME) and diluted with PBS/2-ME to 5 and 50 μg/mL before adding onto the microarray for analysis. The bound rMsgal1-L1 was detected with a goat anti-His IgG labeled with AlexaFluor 488 (Invitrogen). Results were analyzed by GLAD (Mehta and Cummings, 2019). Glycan models were constructed using GlycoGlyph (Mehta and Cummings, 2020).

### Thermal stability, optimal pH for ligand binding, and stability in a non-reducing environment

The thermal stability of purified and recombinant Msgal1-L1 was examined by incubating the samples (100 μL, 3 μg/mL) in PBS/ME at various temperatures for 30 min, cooling on wet ice, and titrating them with human type O red blood cells (RBCs) as previously described (Ahmed et al., 1996). The optimal pH for ligand binding of purified skin Msgal1-L1 in solution was determined by incubating 60 μL of the Msgal1-L1–HRP conjugate in water containing 0.1% Tween 20 (6 μg/mL) mixed with 60 μL of various buffers (0.2 M) at pH ranging from 3.75 to 8.5 for 1 h. The buffers were citrate-phosphate, pH 4.0–6.0; phosphate, pH 6.5–8.0; and carbonate-phosphate, pH 8.5–9.5. After adjusting the pH back to 7.5, 100 μL of each mixture was titrated for binding to ASF in a solid-phase assay as described above (Ahmed et al., 1996). To determine the stability in a non-reducing environment, the purified striped bass galectin (100 μg) was absorbed on 1 mL lactosyl-Sepharose and the matrix was thoroughly washed with aerated PBS (20 mL) and stored at room temperature (RT) for 8 days as previously described (Ahmed et al., 1996). Control matrices contained the same amount of galectin in ME/PBS. At the end of incubation, the bound galectin was eluted with 2 mL of ME/PBS/0.1 M lactose, dialyzed with ME/PBS in the presence of 2 mg of BSA, and tested for the hemagglutinating activity.

### Expression and distribution of Msgal1-L1 in striped bass tissues and epidermal mucus

#### Localization of Msgal1-L1 in striped bass tissues and epidermal mucus

Adult and juvenile striped bass were euthanized by overdose of phenoxyethanol (0.079 ppt) and dissected immediately. Samples of various tissues, including skin, gills, muscle, spleen, liver, and sections of the digestive tract (buccal cavity, esophagus, stomach, pyloric caeca, and intestine) were fixed in neutral buffered formalin (NBF) [100 mL of formalin (37% formaldehyde in distilled water) and 6.5 g of sodium phosphate dibasic (anhydrous)/4.0 g of sodium phosphate monobasic/900 mL of distilled water]. Fixed tissues were sectioned (4 μm) using a Microm HM 340, with Leica 819 and mounted on slides following standard protocols. The slides were incubated with methanol containing 0.3% H_2_O_2_ for 30 min at RT to inactivate endogenous peroxidase, followed by incubation with 2% (w/v) BSA for 1 hour at RT, and blocked using the Avidin/Biotin Blocking Kit (Vector Laboratories) according to the manufacturer’s instructions. To determine Msgal1-L1 expression, the slides were probed with protein A-Sepharose purified rabbit anti-Msgal1-L1 IgG at 1:1,000 dilution for 1 h at RT followed by incubation with biotin-conjugated goat anti-rabbit IgG (VECTASTAIN^®^ Elite ABC Kits, Vector Laboratories) for 30 min at RT and streptavidin-HRP. Purified IgG from rabbit pre-immune serum and blocking buffer only (no primary IgG) served as negative controls. The bound antibody–enzyme complex was visualized using stable DAB (3,3′-diaminobenzidine tetrahydrochloride, 0.0045% H_2_O_2_ in 50 mM Tris-HCl pH 7.5) (Invitrogen). To examine the presence of Msgal1-L1 in mucus, crude mucus was spread on glass slides, and after air-drying, the slides were processed as above.

#### Presence of Msgal1-L1 in head kidney macrophages

Macrophages were prepared following Goetz et al. (2004). Briefly, after fish were euthanized, head kidney was removed, placed in sterile 200-µm nylon mesh bags (Spectrum Laboratories, CA), squeezed through the bag using sterile forceps into 5 mL of Leibovitz’s L-15 medium (GIBCO) supplemented with 5% heat inactivated fetal bovine serum and penicillin (100 U/mL)/streptomycin (100 µg/mL). Cells (1.0 × 10^4^ cells/mL) were placed on polylysine-treated round cover slips placed in wells of a 24-well plate (1 mL/well) and incubated for 24 h at 18°C. Non-adhering cells were removed by washing with PBS three times and supplemented with fresh medium. After 48 h of culture at 18°C, the medium was removed, and cover slips were gently washed with PBS containing 0.9 mM CaCl_2_ and 0.33 mM MgCl_2_ [PBS (+)] three times. Cells were fixed with 2% PFA/PBS (+) for 15 min at RT. After washing with PBS (+) for 5 min six times, cells in one-half of the slides were permeabilized by adding 0.2% Triton-X in PBS (+) to the wells and incubated for 15 min at RT. The remaining half of the slides were tested as “non-permeabilized cells.” Slides were blocked with 5% goat serum/1% BSA in PBS (+) for 1 h at RT. Cover slips were incubated with purified rabbit anti-Msgal1-L1 IgG or pre-immune IgG (600 ng/mL) with 3% goat serum/1% BSA in PBS (+) for 1 h at RT. After washing, cover slips were incubated with Alexa 488-labeled anti-rabbit goat IgG (BD) (1:200) with 1% BSA in PBS (+) for 1 h at RT in the dark. After washing, cover slips were rinsed with Milli-Q water, mounted with VECTOSHIELD Antifade Mounting Medium with DAPI (Vector, H-1200), and fluorescence microscopy images (Microscope Axioplan2, Zeiss) were captured with an DP70 Olympus camera.

### Interaction of rMsgal1-L1 with striped bass skin epidermal mucus

#### Fractionation of epidermal mucus by centrifugation

The crude epidermal mucus was cleared of particulate matter and fractionated by centrifugation at 17,000 rpm for 45 min at 4°C, resulting in three distinct layers: a clear upper layer of lower viscosity, an opaque more viscous layer immediately below that likely contain the highest concentration of mucins, and a loose pellet of solid cellular debris. Fractions obtained from multiple centrifugations were separated and corresponding layers were pooled as “clear upper layer pool” and “viscous intermediate layer pool.”

#### Enzyme treatment of epidermal mucus

Pooled mucus layers were treated with N-glycosidase F [peptide-*N* ^4^-(acetyl-β-glucosaminyl) asparagine amidase], O-glycosidase (O-glycopeptide endo-D-galactosyl-*N*-acetyl-α-galactosamino hydrolase, Roche), endoglycosidase H, or all three enzymes combined. A hundred microliters of mucus sample was added to 0.05 M sodium phosphate buffer (pH 6.0) containing N-glycosidase F (15 mU/mL), O-glycosidase (15 mU/mL), and/or endoglycosidase H (10 mU/mL), respectively, and incubated for 4 h at 37°C.

#### Binding of rMsgal1-L1 to epidermal mucus fractions

Aliquots (100 μL/well) of untreated or glycosidase-treated epidermal mucus pooled layers were dot-blotted onto PVDF membranes using a Bio-Dot Microfiltration Apparatus (Bio-Rad) under vacuum in replicate wells. The blotted membrane was cut into replicate strips for three detection methods: PAS staining to detect carbohydrates, Coomassie Blue to detect total protein, and rMsgal1-L1 to detect the presence of mucus endogenous ligands. PAS staining was performed by washing membranes in three changes of water (1 mL/cm^2^) and transferred to freshly prepared solution of 1% (v/v) periodic acid in 3% (v/v) acetic acid (1 mL/cm^2^) for 30 min at RT. Membranes were rinsed twice in deionized water, transferred to Schiff’s reagent (Sigma) for 30 min (0.5 mL/cm^2^), washed again three times for 2 min each in deionized water, and air-dried. For protein detection, membranes were placed in Coomassie Blue R-250/10% acetic acid/40% methanol in water for 30 min, followed by destaining in 10% acetic acid/40% methanol until background was cleared. For detection of endogenous ligands for Msgal1-L1, the membranes were blocked in 5% BSA/PBS, probing with rMsgal1-L1–HRP with or without lactose (200 mM), and detected with Stable-DAB (Invitrogen).

#### Identification of glycans recognized by Msgal1-L1

Striped bass crude mucus (5–10 mg/mL) was electrophoresed on 4%–20% gradient SDS-PAGE gels at 10 μg per lane, transferred to PVDF membranes. The PVDF membranes were blocked with 5% non-fat milk overnight at RT, and overlaid with 5 μg/mL of biotinylated rMsgal1-L1 to reveal the potential binding of rMsgal1-L1 to striped bass mucus components. Overlays with biotinylated ConA (2.5 μg/mL) were used as controls to reveal the diversity of glycan components in the mucus. In some experiments, to control for carbohydrate binding specificity, prior to overlay onto the PVDF membranes, rMsgal1-L1 was pre-incubated with ASF (200 μg/mL) as a glycosylated inhibitor of galectin binding. The membranes were washed with PBS-T eight times for 5 min each, and binding of rMsgal1-L1 and ConA was detected with streptavidin-conjugated HRP (Pierce) at 1:10,000 in 1% non-fat milk and incubated for 1 h at RT. The membranes were finally washed with PBS-T seven times, developed with the Western Lightening Plus-ECL substrate (PerkinElmer) followed by chemiluminescence assessment by autoradiography at 10 s exposure.

### Interaction of rMsgal1-L1 with bacteria

#### Bacterial cultures

*E. coli* was grown in LB (Difco) broth or agar (1.5% agarose) containing 10 µg/mL kanamycin and incubated at 37°C overnight. *Streptococcus pneumoniae* was cultured as previously described (Nita-Lazar et al., 2015). Briefly, the bacterial culture suspension was streaked on agar plates containing 10% sheep blood and incubated overnight. Single bacterial colonies were inoculated in Todd-Hewitt broth and incubated at 37°C with 5% CO_2_ overnight. *M. marinum* (kindly provided by Dr. Russell Hill, IMET) was streaked on Middlebrook 7H10 agar plates with Dubos Oleic Albumin Complex. The bacterial streaks were inoculated in Middlebrook 7H9 broth and glycerol, enriched with BSA, dextrose, and beef catalase, and cultured in the dark at 30°C for 72 h with continuous shaking (Mustafa et al., 2001). All other bacterial species/strains (*Aeromonas hydrophila, A. veronii, A. trota, Bacillus subtilis, Carnobacterium piscicola, Edwardsiella* sp.*, Pseudomonas aeruginosa, Plesiomonas shigelloides, Photobacterium damselae, Staphylococcus aureus, S. faecalis, V. anguillarum, V. cholerae, V. mimicus, V. parahemolyticus*, and *V. vulnificus*) were cultured on their recommended specific media, as described in the German Collection of Microorganisms and Cell Cultures (Leibniz Institute DSMZ). Bacteria grown in liquid or agar plates were harvested by centrifugation at 4,500 rpm for 15 min, resuspended in their specific liquid medium with 20% glycerol, and stored at –70°C.

#### Agglutination assay

Bacteria stored at –70°C were centrifuged at 300 rpm for 10 min, and the bacterial pellets were resuspended in PBS to 0.5 OD_550_, fixed overnight with 0.05% formaldehyde, washed thoroughly, and resuspended in PBS to 0.5 OD_550_. Agglutination of bacteria by rMsgal1-L1 was tested by mixing 10 μL of rMsgal1-L1 (1 mg/mL in PBS) with 40 μL of bacterial suspensions (0.5 OD_550_) in microtitration plates (U-shaped wells), with overnight incubation at 20°C (Hynes et al., 1990; Dara et al., 2021). Controls consisted of (a) replacing rMsgal1-L1 by 10 μL of PBS and (b) replacing the bacterial suspension by a 0.5% v/v solution of human type O RBC (incubation at 4°C). Results were assessed by visual inspection: a positive result was indicated by a spread carpet of agglutinated bacteria or RBC in the bottom of the wells, whereas a negative result was indicated by a compact dot of bacteria or RBC in the center of the well. Negative results were confirmed by tilting plates at a 45° angle and observing the displacement of the cells from the bottom pellet in the wells to identify any false positives.

#### Microbial glycan microarray

Analysis of the bacterial glycan recognition properties of rMsgal1-L1 was carried out at the NCFG (https://research.bidmc.org/ncfg/microarrays), on version 4.2 of the Microbial Glycan Microarray (MGM) printed with approximately 400 glycans (e.g., capsular exopolysaccharides, O-antigen, and lipopolysaccharide (LPS) derived from species/strains of Gram-positive and Gram-negative bacteria in replicates of six) (Wu et al., 2023). The His-tagged rMsgal1-L1 (1 mg/mL) was dialyzed against PBS containing 10 mM-ME (PBS/2-ME) and diluted with PBS/2-ME to 5 and 50 μg/mL before adding onto the MGM for analysis. The bound rMsgal1-L1 was detected with a goat anti-His IgG labeled with AlexaFluor 488 (Invitrogen).

#### Msgal1-L1 binding to bacteria in a solid-phase assay

Suspensions of *E. coli*, *S. pneumoniae*, and *M. marinum* were diluted to OD_600_ = 1.5 [approximately 5 mg/mL protein concentration as per Nanodrop (ThermoFisher)], diluted to 1:100, dispensed into EIA plates at 200 μL per well, incubated overnight at 4°C, fixed with 1% paraformaldehyde (PFA), and neutralized twice with 0.1% glycine for 1 h, then blocked with 3% BSA for 2 h at RT. The biotinylated rMsgal1-L1 (5 µg/mL) was incubated with lactose (400 mM) or PBS alone for 1 h at RT. The wells with immobilized fixed bacteria and blocked with BSA were washed twice each with PBS and PBS-T and incubated with biotinylated rMsgal1-L1 (with or without lactose) or with PBS alone (with or without lactose) for 1 h at RT. The wells were washed thoroughly with PBS and PBS-T and incubated for 1 h at RT with streptavidin-HRP diluted at 1:20,000 in 1% BSA, then washed and developed with HRP substrate for 15 min at RT with shaking. Binding signals from duplicate wells and from two independent experiments were recorded in a plate reader at 450 nm. The absorbance values of the background [no rMsgal1-L1 (only PBS) with or without lactose] were averaged and subtracted from the absorbance values for rMsgal1-L1 (with or without lactose) with each bacteria species.

## Results

### A “proto” type 15 kDa galectin was purified from muscle, skin, and mucus of striped bass

**Figure 1:**
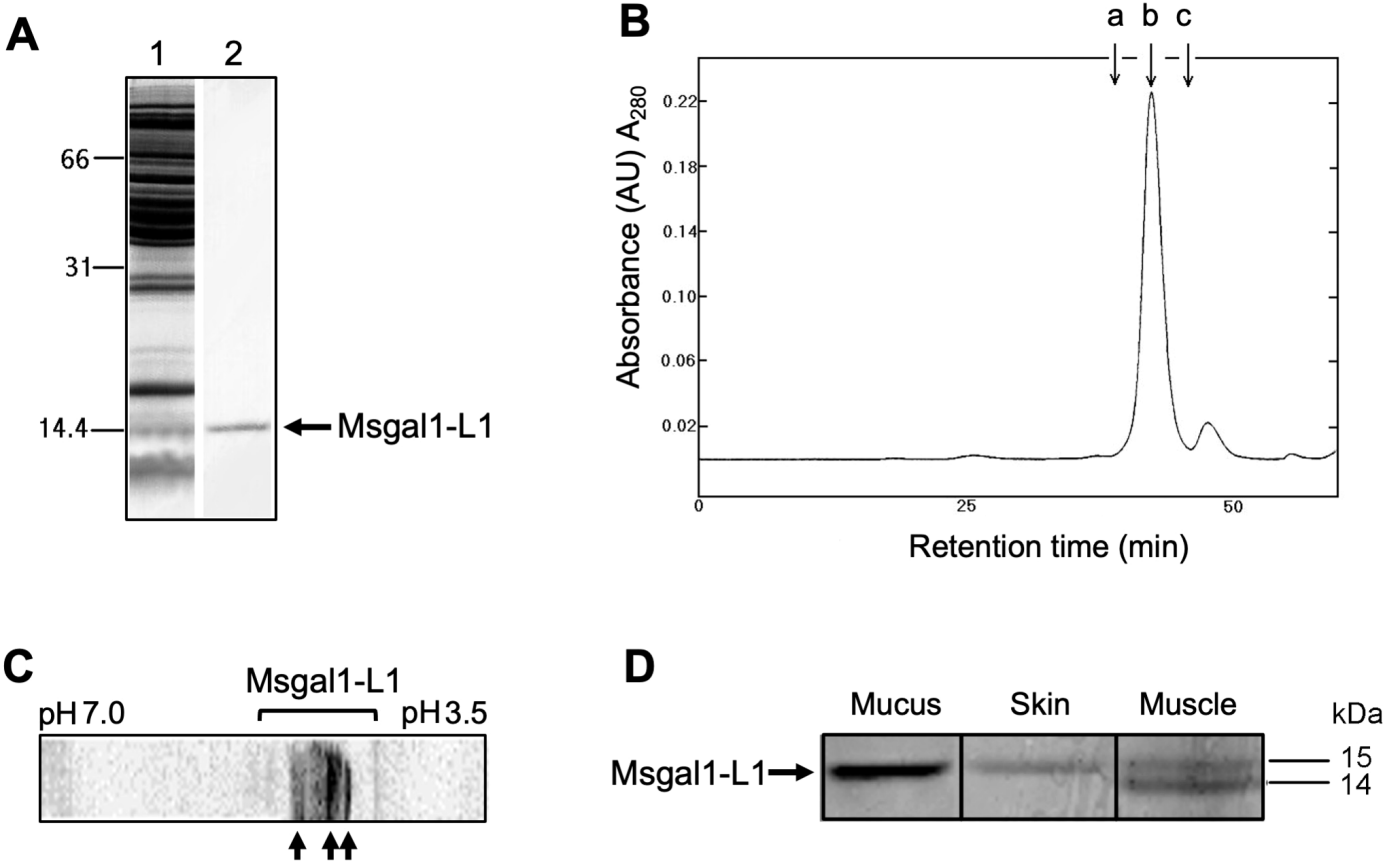
Purification of the proto type galectin Msgal1-L1 from striped bass (*M. saxatilis*). **(A)** SDS-PAGE analysis of Msgal1-L1 purification. Samples were mixed with 2× loading buffer, heated for 1 min prior to loading onto a 15% acrylamide gel. *Lane 1*, striped bass crude skin extract; *lane 2*, lactose eluate from affinity chromatography on lactosyl-sepharose. **(B)** Gel permeation chromatography of Msgal1-L1 on a Superose 6 column. *Arrow a*, peak retention time for BSA (molecular weight: 66,200); *arrow b*, purified Msgal1-L1 dimer (molecular weight: 30,180); *arrow c*, ribonuclease A (molecular weight: 13,700). **(C)** IEF analysis of Msgal1-L1 purified from striped bass skin. Isoelectric focusing (IEF) was run on the range pH 3.5–7.0. The Msgal1-L1 three major isoforms corresponding to isoelectric points (pI) 4.8, 4.9, and 5.2 are indicated by arrows. **(D)** Tricine gel separation of Msgal1-L1 from striped bass epidermal mucus, skin, and muscle. *Lane 1*, mucus galectin; *lane 2*, skin galectin; *lane 3*, muscle galectin. The “doublet” band from muscle tissue contained two proto type galectins (upper band, ~15 kDa; lower band, ~ 14 kDa) that co-purified on affinity chromatography and were successfully separated on a long format 20% tricine gel for N-terminal sequencing.

**Figure 2:**
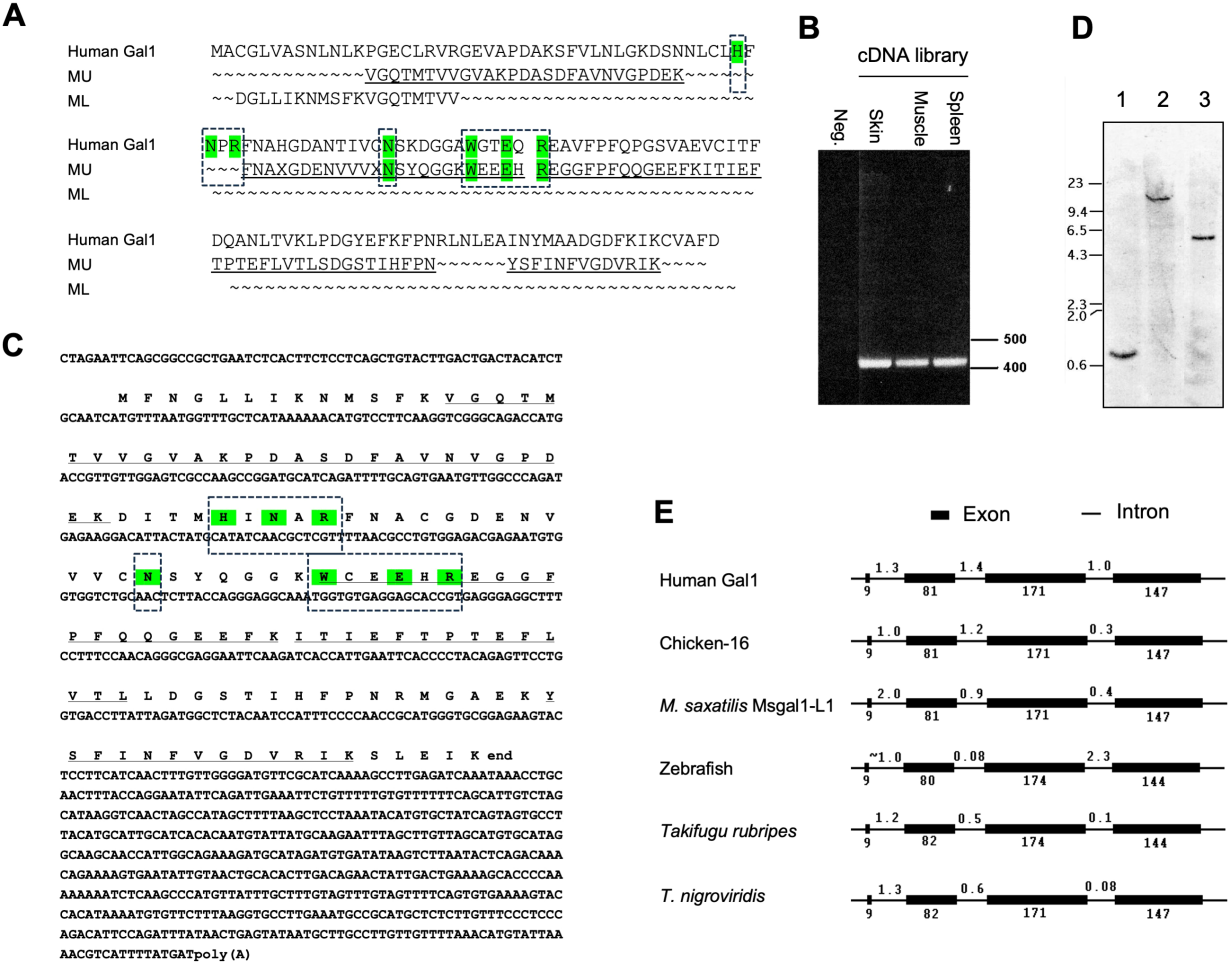
Peptide, transcript, and genomic sequences, and gene organization of Msgal1-L1. **(A)** Alignment of sequenced peptides from muscle “doublet” of striped bass Msgal1-L1 with human galectin-1. Results from peptide digestion and microsequencing of the muscle galectin bands (MU: muscle, upper band; ML: muscle, lower band) were aligned with human galectin-1. “X”s in the sequence were blanks in the sequencing reaction, which often occurs with unmodified Cys in Edman degradation. **(B)** Full-length coding region amplified from striped bass cDNA libraries. cDNA libraries were created using oligo-dT primers, with RNA from skin, muscle, and spleen used as templates. Amplicons were ~425 bp, consistent with the 423-bp predicted length. **(C)** Full-length cDNA and deduced protein sequences of Msgal1-L1. Translation of the full-length cDNA yielded a 134-aa protein that contained all residues from peptide sequence. **(D)** Genomic Southern blot of Msgal1-L1. Striped bass genomic DNA was digested as follows: *lane 1*, BamHI; *lane 2*, XbaI; *lane 3*, SspI. The probe used is the 190-bp PCR product of striped bass galectin exon III. Migration of DNA size markers is indicated on the left (kbp). **(E)** Gene organization of Msgal1-L1 compared to other prototype galectins. The intron/exon organization of the Msgal1-L1 gene is compared to those of other proto type galectins from human, chicken, zebrafish, and two pufferfish species. To simplify the comparison, the 1,600-bp upstream sequence of striped bass galectin is not included. Exons and introns are not to scale. In **(A, C)**, the key amino acid residues that interact with the carbohydrate ligand are highlighted in color and boxed.

Galectins from striped bass skin and muscle tissues and epidermal mucus were purified through a first step of affinity chromatography on lactosyl-Sepharose. After the tissue extracts or diluted epidermal mucus were passed through the column, washes of the resin with high-salt buffer followed by low-salt buffer in the presence of EDTA eliminated any non-specifically binding proteins, as well as calcium-dependent lactose-binding proteins. The remaining bound protein that was eluted from the column by 0.1 M lactose appeared in a single peak with a yield of 77 μg/kg of skin wet tissue. The specific activity of the purified protein was 5 × 10^2^ mg/mL as assessed by agglutination of human type O erythrocytes, with an 11,000-fold purification from the skin crude extract (data not shown). The yield of the purified protein from muscle tissue was 15 μg/kg following a similar purification protocol. On SDS-PAGE under reducing conditions, the lactose eluate from skin extracts appeared as a single band corresponding to a molecular weight of 15 kDa and was designated as Msgal1-L1 (*M. saxatilis* galectin1-like protein 1) (**Figure 1A**). The molecular weight of the native Msgal1-L1 was approximately 30 kDa as estimated from gel permeation chromatography (**Figure 1B**). Despite the apparent subunit size homogeneity observed by SDS-PAGE, analysis of the purified Msgal1-L1 from skin by IEF showed clear heterodispersion, with the protein resolved in at least three major isoforms (isolectins) corresponding to isoelectric points (pI) 4.8, 4.9, and 5.2, indicated by arrows in **Figure 1C**, with minor components appearing as a smear. As observed with most proteins (Wu et al., 2021), this charge heterogeneity is due to point replacements in ionizable amino acids along the peptide sequence that determine the protein net charge, mostly without major structural consequences. Interestingly, the purified protein from various preparations of muscle extract consistently revealed an additional lower band approximately at 14.5 kDa (**Figure 1D**). The lower band was confirmed to be a truncated form of Msgal1-L1 (see below and **Figure 2A**).

### Msgal1-L1 harbors a single canonical galectin-like carbohydrate recognition domain

Amino acid sequences of four peptides resulting from the trypsin cleavage of the Msgal1-L1 protein (**Figure 2A**) enabled the design of degenerate primers, which upon PCR amplification from either skin, muscle, or spleen cDNA, resulted in a PCR product of approximately 190 bp. Cloning and sequencing of the PCR product resulted in predicted amino acid sequences that matched the amino acid sequence of the tryptic peptides. To obtain a full-length cDNA corresponding to Msgal1-L1, specific primers were designed and 5′- and 3′-RACE reactions were performed from the cDNA library. The entire cDNA for Msgal1-L1 was 992 bp long, with an open reading frame (ORF) 405 bp in the coding region (**Figures 2B, C**). The ORF encoded a 135-amino acid (aa) peptide with a calculated molecular weight of 14,983 Da, in agreement with the apparent molecular weight observed on SDS-PAGE. The continuity of 5′- and 3′- RACE was confirmed by sequencing the amplicon generated with the primers sbfullf1 and sbfullr1 from each cDNA library generated from skin, muscle, and spleen tissues (**Figure 2B**). The predicted pI for the Msgal1-L1 protein is 5.15, which is consistent with the observed pI in the IEF analysis (**Figure 1C**).

The amino acid sequence of Msgal1-L1 contains a single canonical galectin-like CRD and, as described for most galectins, there was no predicted secretion signal or transmembrane domain found in the peptide sequence (**Figure 2C**). Overall, the primary structure of Msgal1-L1 shows close similarity with the mammalian proto type Gal-1 (**Figures 2A, C**). Most importantly, analysis of the predicted amino acid sequence of the CRD region revealed that the galectin CRD of Msgal1-L1 contains all seven invariable residues (H^44^, N^46^, R^48^, N^61^, W^68^, E^71^, and R^73^; residue numbers are those of bovine spleen Gal-1) that are recognized as essential for carbohydrate binding activity in the mammalian proto type galectins (**Figure 2C**).

### The structural organization of the Msgal1-L1 gene is consistent with that of mammalian galectin genes

Southern blot analysis of the striped bass genomic DNA following restriction digest revealed a single copy gene detected by a 200-bp probe directed at Exon III of *MSGAL1-L1*, which encodes the conserved residues of the carbohydrate binding site of proto type galectins (**Figure 2D**). The stringency used for this blot should have potentially detected any other galectin genes that were within 40% identity. The intron/exon analysis of the Msgal1-L1 gene sequence revealed that its organization is similar to that of human, mouse, and chicken prototype galectins, as well as to galectins from other fish species (**Figure 2E**). There are four exons and three introns, all with canonical exon/intron boundaries of GT/AC. The sizes of the exons are 9, 81, 171, and 147 bp for exons I–IV, respectively. The sizes of the introns are 2,000, 871, and 425 bp for introns I–III, respectively. The genomic amplicon from the start of exon I to the predicted stop codon in exon IV yielded a product of approximately 3,700 bp, which matched the predicted gene size. This amplicon also yielded products of predicted sizes with nested primers. Analysis of the upstream regions of *MSGAL1-L1* by automated searches for transcription factors identified numerous potential control elements. The same search performed on the introns suggested the presence of control elements in intron I. The sequence downstream of the predicted stop codon, both transcribed (500 bp) and non-transcribed (500 bp genomic beyond poly A signal), revealed a predicted polyadenylation signal and a weak T/GT-rich region.

### The phylogenetic analysis of Msgal1-L1 revealed close relationships with galectins from teleost fish

**Figure 3:**
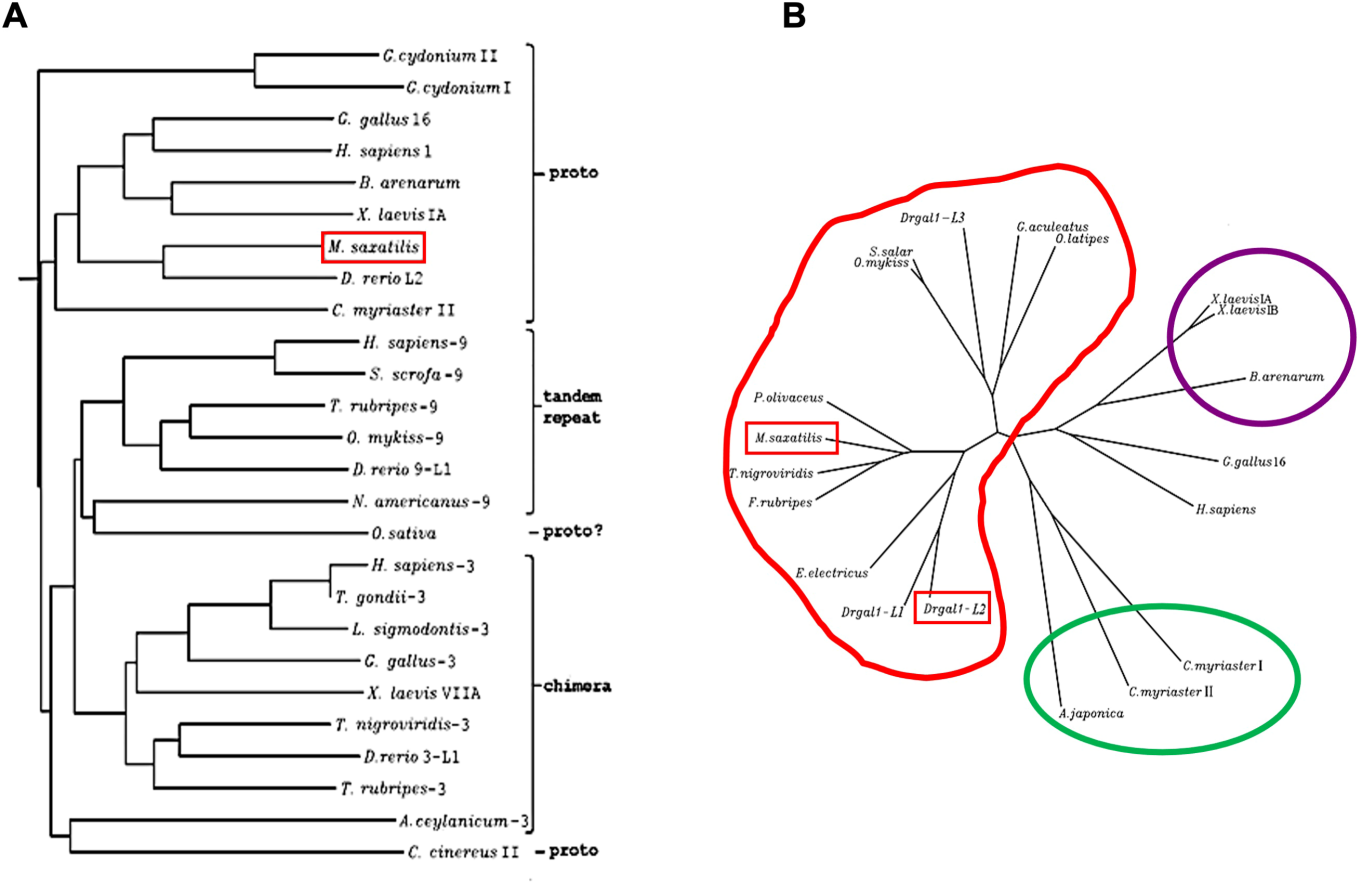
Phylogenetic analysis of Msgal1-L1. **(A)** Neighbor joining tree of Msgal1-L1 and prototype galectins from mammals, birds, fish, and tunicates. The phylogenetic tree generated using the ClustalW program included genome sequences and cDNA sequences representative of prototype galectins from mammals, birds, fish, and tunicates, deposited in GenBank and in the genome databases at TIGR and HGMP Resource Centre (Fugu Genomics Group). The ClustalW program uses the NJ (Neighbor Joining) method of Saitou and Nei (1987). **(B)** Clusters of galectin sequences identified through the phylogenetic analysis of Msgal1-L1. Msgal1-L1 clusters closely with flounder, green-spotted pufferfish, and Japanese pufferfish. Slightly distant are the electric eel and two of three zebrafish prototype galectins. Galectins of salmonids, stickleback fish, and the anguillids are as distant as the mammalian and bird proto type galectins.

The NJ phylogenetic tree demonstrated a close relationship among all of the proto type galectins (**Figure 3A**). The unrooted tree, however, revealed that Msgal1-L1 clusters with galectins from several other teleost species, and this group is distant from the tight group formed by mammals, birds, and amphibians (**Figure 3B**). Specifically, Msgal1-L1 clusters closely with galectins from bastard flounder (*Paralichthys olivaceus*), green-spotted pufferfish (*Dichotomyctere nigroviridis*), and the Japanese pufferfish (*Takifugu rubripes*). Slightly more distant are the galectin from electric eel (*Electrophorus electricus*) and two of three zebrafish (*Danio rerio*) proto type galectins. The cluster comprising proto-type galectins from salmonids, stickleback fish, and the anguillids is as distant from that of Msgal1-L1 as those from mammalian and bird proto type galectins (**Figure 3B**).

### The homology model of Msgal1-L1 showed a structure similar to the zebrafish proto type galectin Drgal1-L2

**Figure 4:**
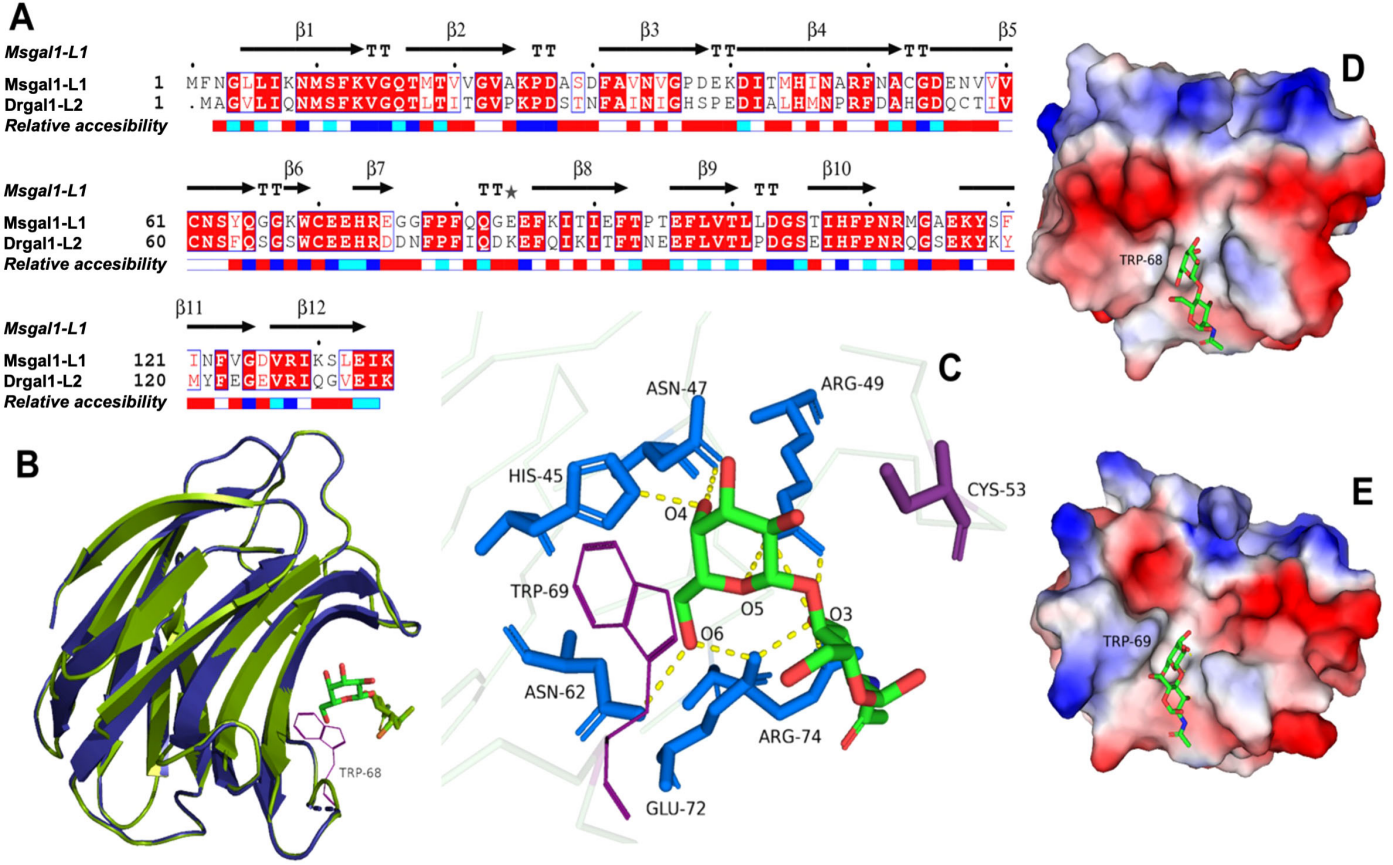
Homology model of Msgal1-L1 based on crystal structure of the *D. rerio* galectin-1-L2 (DrGal1-L2, PDB ID: 6E20). **(A)** Alignment of Msgal1-L1 with zebrafish Drgal1-L2. The alignment shows the protein sequence of Msgal1-L1 with zebrafish Drgal1-L2 used as the template. **(B)** Overlay of Msgal1-L1 with Drgal1-L2. Overlay of the Msgal1-L1 model with the Drgal1-L2 crystal structure shows the interaction of the galectin CRD with the glycan ligand N-acetyl-D-lactosamine (LacNac; Galβ1,4GlcNAc). **(C)** Interactions of amino acids in the Drgal1-L2 CRD with the glycan ligand. The detailed interactions of key amino acid residues in the Drgal1-L2 CRD with LacNAc. **(D)** Electrostatic surface of Drgal1-L2. The electrostatic surface distribution (red: negative; blue: positive) of the Drgal1-L2 monomer with bound LacNac. **(E)** Electrostatic surface of Msgal1-L1. The electrostatic surface distribution (red: negative; blue: positive) of the Msgal1-L1 monomer with bound LacNac.

The structure of Msgal1-L1 was modeled based on the Drgal1-L2 structure as a template, which showed 60.61% identity with target sequence Msgal1-L1 (**Figure 4A**), the highest sequence identity among the 316 potential templates examined. For example, Msgal1-L1 showed 39.53% and 34.33% identity with the bovine Gal-1 [Protein Data Bank (PDB) ID: 1SLB] and the Gal-1 from toad *Bufo arenarum* ovary (PDB ID: 1GAN), respectively. For modeling, we used the SWISS-MODEL template library (SMTL version 2019, PDB release 2019) searched with BLAST (Camacho et al., 2009) and hidden Markov models (HMMs; Remmert et al., 2012) for evolutionarily related structures matching the target sequence. For details on the template search, see the Experimental Procedures section.

**Figure 5:**
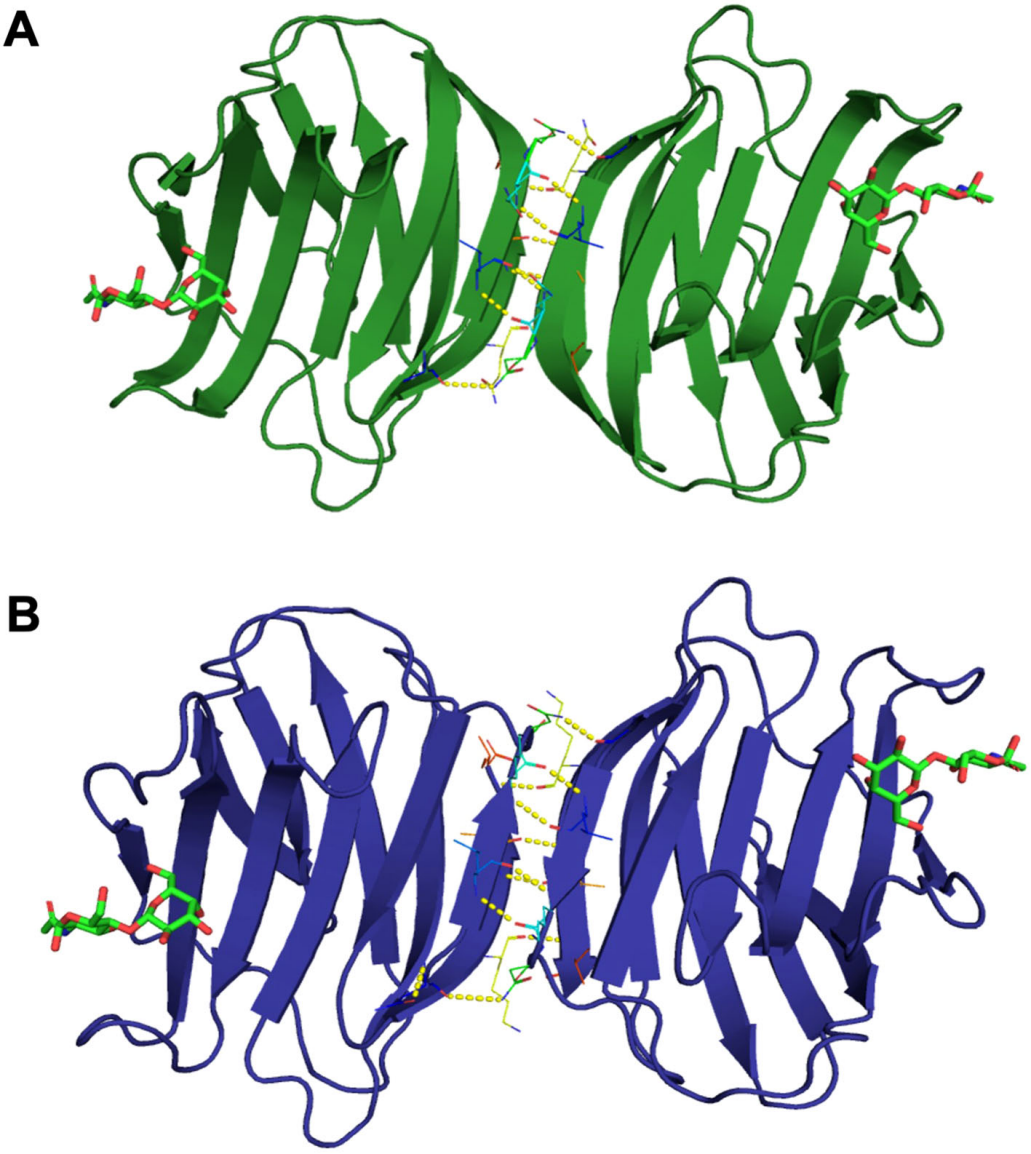
Hydrogen bond interactions at the dimer interface of zebrafish and striped bass galectins. **(A)** Zebrafish galectin Drgal1-L2. The structure of the zebrafish (*Dario rerio*) galectin Drgal1-L2 homodimer (PDB ID: 6E20) in complex with the glycan ligand LacNAc, showing the hydrogen bond pattern at the dimer interface (represented by yellow dashes). **(B)** Striped bass galectin Msgal1-L1. The model of striped bass (*Morone saxatilis*) galectin MsGal15 homodimer, showing the hydrogen bond pattern at the dimer interface (represented by yellow dashes).

The final model of the Msgal1-L1 was generated using Drgal1-L2-PDB ID 6E20 (Ghosh et al., 2019) as template and based on the sequence alignment has 95.37% of its residues in the allowed region of the Ramachandran plot and an RMSD between model and template of 0.08 Å for 127 aligned Cα atoms. Based on this model, no differences were observed in the coordination of LacNAc, the glycan ligand preferred by most proto type galectins, between the model and template (**Figures 4B, C**); i.e., it interacts with H^45^, N^47^, R^49^, N^62^, E^72^, and R^74^ as in the template DrGal1-L2—PDB ID 6E20 (Ghosh et al., 2019), except that it differs in the C substitution instead of H^53^. This substitution explains the difference in overall charge distribution between DrGal1-L2 (**Figure 4D**) and the model of Msgal1-L1 (**Figure 4E**). Despite the surface charge differences, the hydrogen bond interaction patterns represented by yellow dashes at the dimer interface of zebrafish DrGal1-L2 in complex with LacNAc (**Figure 5A**) are highly similar to those revealed in the striped bass Msgal1-L1 model (**Figure 5B**).

### Biochemical characterization Msgal1-L1

Given the heterodispersity of the native Msgal1-L1, to enable studies on a reproducible form of the protein, rMsgal1-L1 was produced using the sequence of the single transcript cloned and sequenced as described above. Most subsequent studies aimed at protein characterization were carried out in parallel comparing the purified native Msgal1-L1 and rMsgal1-L1.

#### Expression of recombinant Msgal1-L1

Transformation of BL21(DE3) cells with the Magal1-L1-pET 30 followed by IPTG induction resulted in abundant expression of rMsgal1-L1, which was purified in one step by affinity chromatography on lactosyl-Sepharose. Cleavage of protein tags introduced by the vector was accomplished by incubating lactosyl-Sepharose-bound rMsgal1-L1 with recombinant enterokinase (rEK) for 16 hours, washing to remove enzyme and cleaved tags, and then eluting rMsgal1-L1 with lactose (**Supplementary Figure 1**). Following cleavage with rEK, rMsgal1-L1 showed identical electrophoretic mobility as the native Msgal1-L1 in SDS-PAGE (15% acrylamide) (**Supplementary Figure 1**). The yield of active rMsgal1-L1 was 50–60 mg/L of culture.

#### The thermal stability, optimal pH for ligand binding, and susceptibility to oxidation of Msgal1-L1 is consistent with galectins from vertebrates

**Figure 6:**
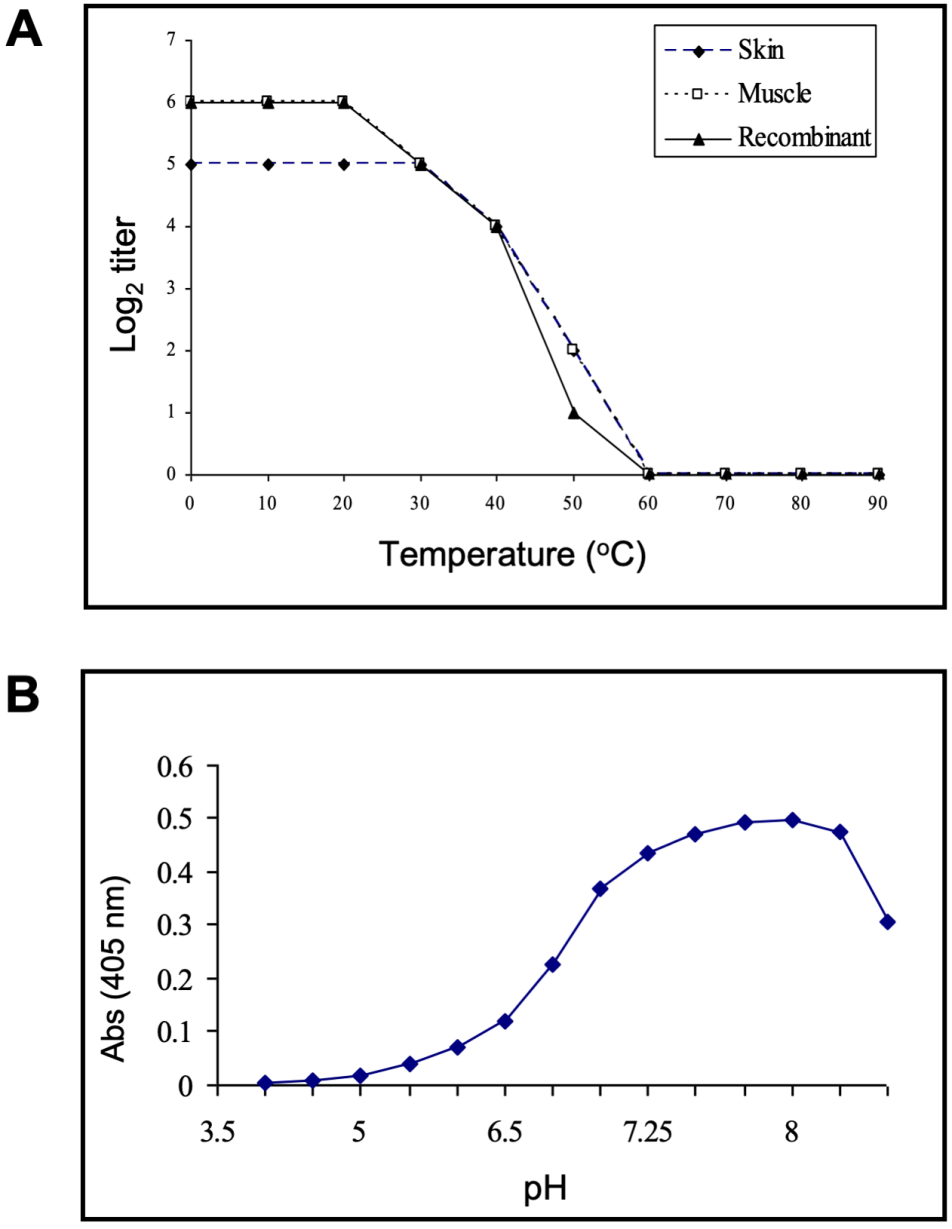
Thermal stability and optimal pH for ligand binding of Msgal1-L1. **(A)** rMsgal1-L1 (3 μg/mL) was incubated in ME/PBS at temperatures ranging from 0°C to 100°C for 30 min, cooled on wet ice, and titrated with human type O erythrocytes (Vasta and Marchalonis, 1986). **(B)** Msgal1-L1–HRP conjugate (6 μg/mL) in 60 μL of water containing 0.1% Tween 20 was mixed with 60 μL of various buffers (0.2 M) at pH ranging from 3.75 to 8.5, and incubated for 1 **(h)** After adjusting the pH back to 7.5, 100 μL of each mixture was titrated for binding to ASF in a solid phase assay as described above (Ahmed et al., 1996).

The stability of the purified Msgal1-L1 from both skin and muscle, and the recombinant equivalent (rMsgal1-L1), was dependent on temperature as measured by hemagglutination activity (**Figure 6A**). From 30°C to 60°C, there is a steady decline in binding activity, with no activity observed above 60°C for either skin or muscle native galectins, as well as the rMsgal1-L1. The binding activity of the native Msgal1-L1 purified from skin was also dependent on the pH of the incubation buffer and showed optimal binding at pH 7.75–8.25 (**Figure 6B**). A preliminary study showed that rMsgal1-L1 in PBS was gradually inactivated in an oxidative environment, as compared to the stable binding activity of the protein in ME/PBS. rMsgal1-L1 could be protected from oxidative inactivation by the presence of excess carbohydrate ligand (50 mM lactose) as previously reported for the toad galectin (Ahmed et al., 1996).

#### Analysis of the carbohydrate specificity of Msgal1-L1 revealed a preference for LacNAc moieties

The carbohydrate specificity of Msgal1-L1 was analyzed by two different approaches: (1) a solid-phase binding-inhibition assay to test a comprehensive collection of mono- and oligosaccharides as inhibitors of binding of Msgal1-L1 to immobilized ASF, and (2) interrogating a glycan microarray for Msgal1-L1 binding.

#### Solid-phase binding-inhibition assay

The carbohydrate-binding specificity of Msgal1-L1 was determined by analyzing the binding of the rMsgal1-L1–HRP conjugate to ASF in the presence of several saccharides in a solid-phase assay under the optimal conditions of pH and temperature established above (pH 8.0; 23°C) and under a reducing environment (10 mM ME) (Ahmed et al., 1996, 2002). For each test saccharide, a complete inhibition curve was obtained, and the molar concentrations that inhibited the binding of the lectin conjugate to ASF by 50% (*I*_50_) were calculated and normalized with respect to Galβ1,4Glc (lactose), which was included in each plate as a standard (**Table 2**). The binding-inhibition profile for Msgal1-L1 was generally similar to those for proto type galectins from zebrafish (*D. rerio*), toad (*B. arenarum*), and domestic cow (*Bos taurus*). For Msgal1-L1, the inhibitory effectiveness of Galβ1,4GlcNAc (*N*-acetyllactosamine; LacNAc) was approximately 3-fold better than lactose, suggesting that additional interactions are established between the protein and the *N*-acetyl group of the disaccharide, making LacNAc the disaccharide unit preferred by Msgal1-L1, as observed with most proto type galectins. In contrast, Galβ1,6GlcNAc was approximately 3- and 4-fold less effective than lactose suggesting that the β1,4 anomeric linkage is strongly preferred to the β1,6 linkage. The presence of a fucose unit linked in α1,2 to the non-reducing Gal moiety decreased the inhibitory effectiveness in about one-third as compared to lactose, most likely by steric hindrance of the protein–Gal interaction. In contrast, the presence of an MeO-2 group on the same non-reducing Gal moiety increased the inhibitory effectiveness in approximately 3-fold, suggesting that additional interactions between the hydrophobic group and the protein CRD are established. Thiodigalactoside (Galβ1S1βGal) was approximately 10-fold better than lactose and approximately 150-fold better than the monosaccharide D-galactose, respectively, indicating that as observed in the crystal structure of the *B. arenarum* proto type galectin–thiodigalactose conjugate, additional interactions are established between the protein and the S and both Gal groups (Bianchet et al., 2000). A hydrophobic phenyl group linked to the reducing end of Galβ1,3GlcNAc strengthened the Msgal1-L1 interaction with the ligand 6.5-fold.

**Table 2:** Binding inhibition of Msgal1-L1 by carbohydrates, compared with other proto type galectins.

	Relative inhibitory activity			
Carbohydrate	*Morone saxatilis*	*Danio rerio*	*Bufo arenarum*	*Bos taurus*
GalNacβ1,3Glc	2.8	—–	4.45	—–
Fucα1,2Galβ1,4Glc	0.33	0.35	0.17	0.36
Gal	0.007	0.004	0.005	0.0079
Galβ1,3Ara	4.10	2.1	1.28	2.56
Galβ1,4Fru*_f_*	1.9	1.0	1.0	—–
Galβ1,4Glc	1.0	1.0	1.0	1.0
Galβ1,4Glcβ(1-0)Me	1.74	2.9	2.14	1.33
Galβ1,4Glcβ-OPhNH_2_ (p)	2	1.4	1.71	1.48
Galβ1,4GlcNac	3.07	7.9	3.75	5.54
Galβ1,4Man	1.74	1.5	2.5	2.34
Galβ1,6GlcNac	0.225	0.03	0.83	0.12
Galβ1-3GlcNacβ-OPhNO_2_ (p)	6.5	2.3	1.78	4.5
Galβ1S1βGal	10.0	12.2	3.9	9.0
Galβ-OMe	0.009	0.004	0.006	0.0044
Galα1,6Glc	0.019	0.004	0.0075	0.0053
Galα-OMe	0.027	0.006	0.03	0.01
MeO-2Galβ1,4Glc	3.33	2.9	2.5	4.08

A carbohydrate-inhibition solid-phase assay was carried out as described in Experimental Procedures to examine the carbohydrate specificity of Msgal1-L1 from striped bass (*Morone saxatilis*) and to compare the inhibition profiles with those for proto type galectins from zebrafish (*Danio rerio*), toad (*Bufo arenarum*), and domestic cow (*Bos taurus*).

#### Glycan microarray analysis of the carbohydrate specificity of Msgal1-L1

**Figure 7:**
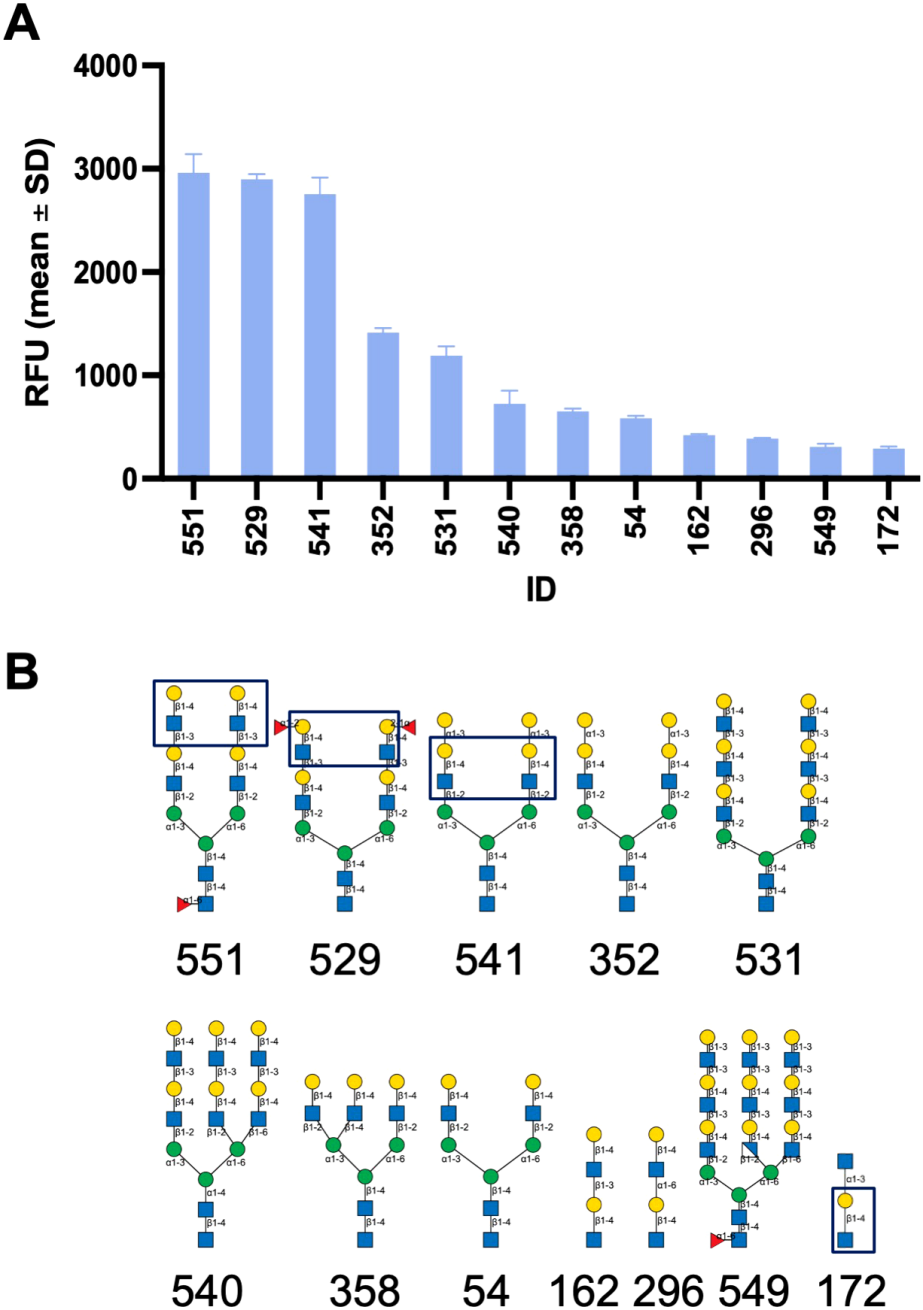
Glycan microarray analysis of the carbohydrate specificity of Msgal1-L1. After dialysis against PBS/ME, rMsgal1-L1 (1 mg/mL) was diluted with PBS/ME to 5 and 50 μg/mL before adding onto the microarray for analysis. Results were analyzed by GLAD (Mehta and Cummings, 2019, *Bioinformatics*) [Bioinformatics; https://doi.org/10.1093/bioinformatics/btz075]. **(A)** The top 12 oligosaccharides based on relative fluorescence units (RFU) are plotted in decreasing order of binding of Msgal1-L1 (5 μg/mL). The RFU values for the oligosaccharides 1–12 illustrated are as follows (glycan microarray code number in parentheses): 1 (551), 2,963 ± 180; 2 (529), 2,897 ± 52; 3 (541), 2,755 ± 160; 4 (352), 1,416 ± 41; 5 (531), 1,193 ± 89; 6 (540), 724 ± 127; 7 (358), 651 ± 28; 8 (54), 584 ± 23; 9 (162), 420 ± 13; 10 (296), 387 ± 9; 11 (549), 309 ± 29; 12 (172), 291 ± 20. **(B)** The structures of the top 12 structurally related oligosaccharides recognized in the microarray by Msgal1-L1 in the same order as in **(A)** revealed the preference of Msgal1-L1 for terminal and internal LacNAc moieties (boxed in selected structures for illustration). Glycan models were constructed using GlycoGlyph (Mehta and Cummings, 2020, Bioinformatics) [Bioinformatics; https://doi.org/10.1093/bioinformatics/btaa190].

Glycan microarray analysis carried out at the NCFG revealed the carbohydrate specificity of Msgal1-L1. The top 12 oligosaccharides based on relative fluorescence units (RFU) plotted in decreasing order of binding of Msgal1-L1 (5 μg/mL) are illustrated in **Figure 7A**. The RFU values for the oligosaccharides 1–12 are as follows (glycan microarray code number is in parentheses): 1 (551), 2,963 ± 180; 2 (529), 2,897 ± 52; 3 (541), 2,755 ± 160; 4 (352), 1,416 ± 41; 5 (531), 1,193 ± 89; 6 (540), 724 ± 127; 7 (358), 651 ± 28; 8 (54), 584 ± 23; 9 (162), 420 ± 13; 10 (296), 387 ± 9; 11 (549), 309 ± 29; 12 (172), 291 ± 20. The structures of the top 12 oligosaccharides recognized in the microarray by Msgal1-L1 illustrated in the same order as above (**Figure 7A**) revealed the preference of Msgal1-L1 for terminal and internal LacNAc moieties (boxed in selected structures for illustration) (**Figure 7B**), thereby confirming the results of the binding-inhibition experiments (**Table 2**) described above.

### Msgal1-L1 is present in macrophages and epithelial- and fibroblast-like cells from mucosal tissues and epidermal mucus of striped bass

Immunohistochemical analysis of skin and gill sections with the anti-Msgal1-L1 antibody showed intense staining of the surface epithelial layer of the epidermis, as well as the dermis below, particularly the connective tissue. Furthermore, strongly stained macrophage- or fibroblast-like cells were observed in all sections examined from skin and gills (**Supplementary Figure 2**). Similar positive signals were also observed in sections of lip, tongue, the buccal cavity, and various layers of selected sections of the digestive tract that display a mucosal surface, including esophagus, stomach, pyloric caeca, and proximal intestine (not shown). In microvilli of the pyloric caeca, Msgal1-L1 localized in a cell type with a macrophage- or fibroblast-like morphology, but none was observed on the epithelial cells lining the lumen (**Supplementary Figure 3**). Most notably, the mucus-producing goblet cells that were clearly identifiable in these sections showed no stain at all.

**Figure 8:**
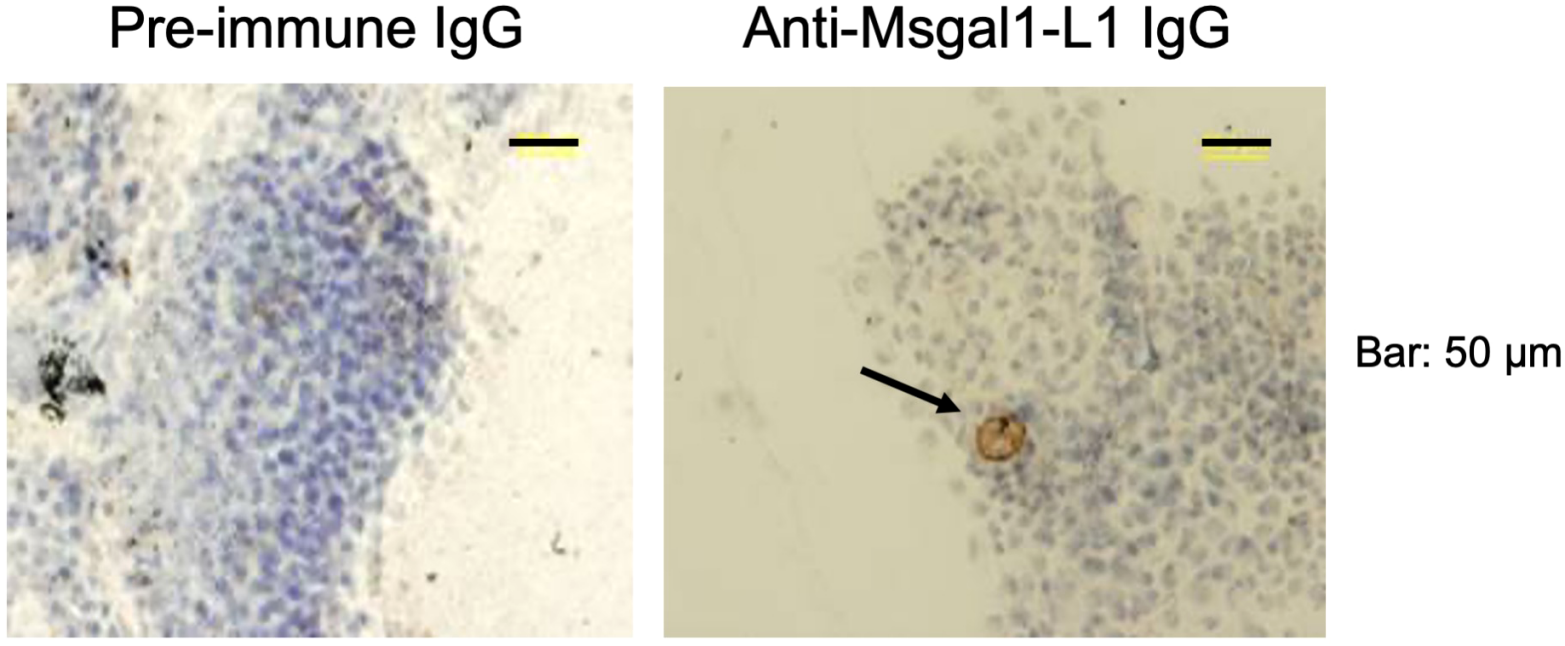
Localization of Msgal1-L1 in macrophage-like cells in epidermal mucus. Crude epidermal mucus was collected, fractionated by centrifugation, and the loose pellet was spread on glass slides and allowed to air-dry. Slides were fixed and stained with hematoxylin–eosin and probed for the presence of Msgal1-L1 with rabbit anti-Msgal1-L1 IgG (with pre-immune rabbit IgG as control), followed by biotin-conjugated goat anti-rabbit IgG (VECTASTAIN^®^; 30 min at RT), as described in Experimental Procedures. Bound antibody was detected with Stable DAB.

**Figure 9:**
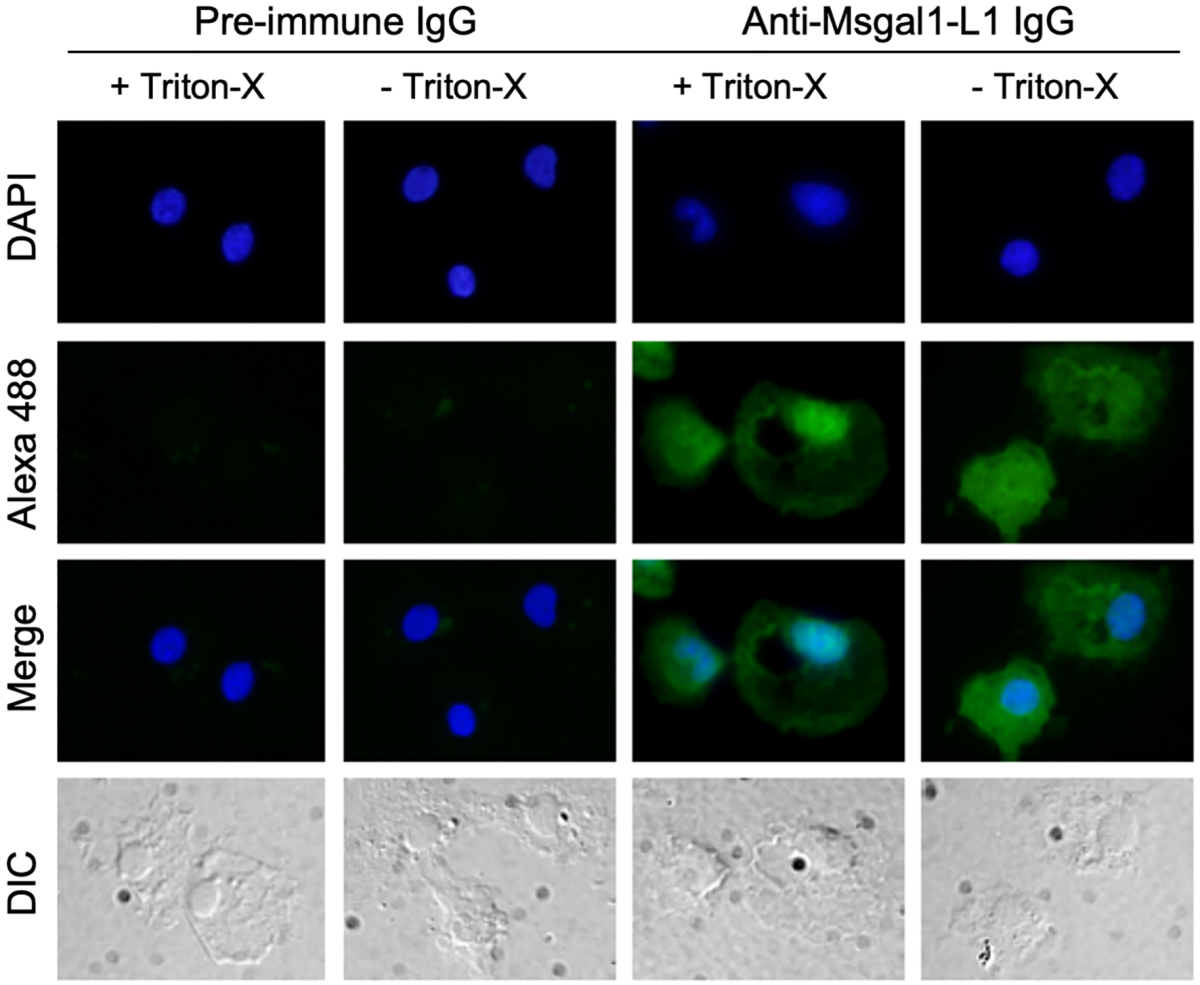
Localization of Msgal1-L1 in macrophages isolated from head kidney. Striped bass macrophages were isolated (Goetz et al., 2004) from head kidney of euthanized fish, and adhered to polylysine-treated cover slips as described in Experimental Procedures. Cells were either permeabilized with 0.2% Triton-X in PBS (+) (+ Triton-X) or untreated (− Triton-X). Presence of Msgal1-L1 in both permeabilized and non-permeabilized cells was detected with rabbit anti-Msgal1-L1 IgG or pre-immune rabbit IgG as control, followed by Alexa 488-labeled anti-rabbit goat IgG (1:200) with 1% BSA in PBS (+) as described in Experimental Procedures. Images were captured by a camera (DP70, Olympus) connected to a fluorescence microscope (Axioplan2, Zeiss). Differential interference contrast (DIC) images were also captured.

Analysis of crude epidermal mucus showed that Msgal1-L1 was concentrated in the periphery of macrophage-like cells that exhibited a distinct morphology and were much larger than the numerous epithelial cells sloughed from the epidermis (**Figure 8**). In some mucus samples, a light diffuse Msgal1-L1 staining was observed in the amorphous mucus matrix. In contrast with mucosal tissues at the interphase with the external environment such as skin and gills, internal organs such as liver and brain showed positive signals for Msgal1-L1 only in cell clusters in the lumen of blood vessels (not shown). To confirm the potential localization of Msgal1-L1 in striped bass macrophages, homogeneous populations of these cells were isolated from head kidney, an important immune organ in teleost fish, and examined by immunohistochemical analysis for the presence of Msgal1-L1. Both permeabilized and non-permeabilized cells showed strong positive reaction, indicating that Msgal1-L1 is present not only in the macrophage cytoplasm but also on the cell surface (**Figure 9**). In all sections observed, the pre-immune IgG control showed no positive signals.

### Msgal1-L1 binds to selected glycosylated components of striped bass epidermal mucus

Mucus collection yielded approximately 20 mL of mucus/fish. The mucus was fractionated by centrifugation, yielding three distinct layers: a clear top layer, an opaque viscous layer below, which was assumed to contain the highest concentration of soluble mucins, and an insoluble pellet, which was shown as intact cells and cell and amorphous debris through microscopic examination. The potential binding of Msgal1-L1 to glycosylated components in the striped bass epidermal mucus was analyzed on PVDF membranes onto which the untreated and glycosidase-treated mucus layer fractions described above had been immobilized by dot-blotting, followed by overlay of biotinylated rMsgal1-L1 onto the PVDF membrane.

#### Binding of Msgal1-L1 to untreated and glycosidase-treated epidermal mucus in dot blot

**Figure 10:**
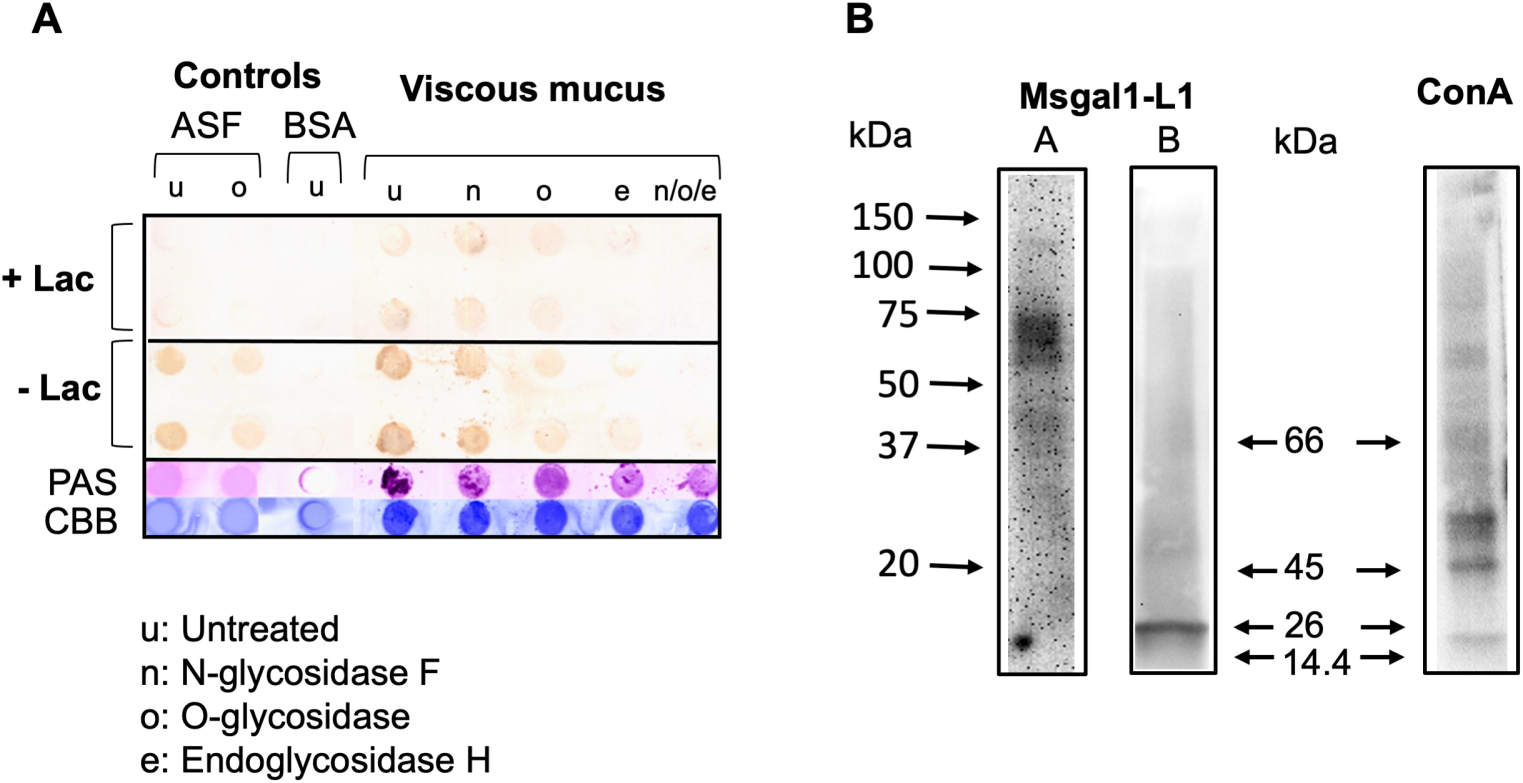
Binding of Msgal1-L1 to striped bass epidermal mucus glycans. **(A)** Upon fractionation by centrifugation, the viscous intermediate layer of striped bass crude mucus either was kept untreated (u) or was treated with N-glycosidase F (n), O-glycosidase (o), or endoglycosidase H (e), and with all three enzymes combined (n/o/e), as described in Experimental Procedures. Samples of untreated and glycosidase-treated viscous fraction were dot-blotted on PVDF membranes and probed with rMsgal1-L1 with and without lactose. PAS and CBB (Coomassie Brilliant Blue) stains were used to assess the relative proportions of carbohydrate and protein in each dot. ASF (asialofetuin) and BSA (bovine serum albumin) were used as glycosylated and non-glycosylated controls, respectively. **(B)** Identification of glycans recognized by Msgal1-L1: Striped bass epidermal mucus (5–10 mg/mL) from healthy **(A)** or *M. marinum*-infected fish **(B)** was electrophoresed on 4%–15% gradient SDS-PAGE gels at 10 μg per lane and transferred to PVDF membranes. Binding of Msgal1-L1 to mucus components was examined by overlaying the membrane with 5 μg/mL of biotinylated Msgal1-L1. Overlays with biotinylated ConA (2.5 μg/mL) were used as controls. Binding of both Msgal1-L1 and ConA was detected using HRP-conjugated streptavidin (Pierce) and developed using Western Lightening Plus-ECL reagent (PerkinElmer).

The biotinylated rMsgal1-L1 strongly bound to the untreated viscous mucus layer, suggesting that mucus components, most likely mucins, display abundant non-reducing terminal galactosyl moieties that are recognized and bound by Msgal1-L1 (**Figure 10A**). The clear mucus fraction showed a similar binding pattern, but with much less intense signals (not shown). Binding of rMsgal1-L1 to the N-glycosidase F-treated mucus was only partially reduced, suggesting that only a limited portion of the mucus oligosaccharides are N-linked. Binding of rMsgal1-L1 to either the O-glycosidase-treated or endoglycosidase-H-treated mucus layers drastically reduced the intensity of the signals, and more so when they had been treated with the three enzymes, N-glycosidase F, O-glycosidase, and endoglycosidase-H. Pre-incubation of rMsgal1-L1 with lactose (200 mM), an inhibitory sugar for Msgal1-L1 binding, substantially reduced Msgal1-L1 binding to the untreated and glycosidase-treated mucus layers, revealing that interactions of Msgal1-L1 with epidermal mucus components are protein–carbohydrate in nature and sugar-specific. The inhibition of Msgal1-L1 binding to mucus components by lactose, however, was not complete, suggesting that Msgal1-L1 recognizes Gal-displaying mucus oligosaccharides with affinity high enough that cannot be overcome by the lactose concentration used.

#### Identification of mucus components recognized by Msgal1-L1

To obtain information about the electrophoretic mobility of glycosylated components recognized by Msgal1-L1 in striped bass epidermal mucus, mucus was examined by a galectin overlay assay. For this, whole mucus was electrophoresed on polyacrylamide gradient gels and transferred to PVDF membranes, which were overlaid with biotinylated rMsgal1-L1 (5 mg/mL), or biotinylated ConA as controls. Representative results of the rMsgal1-L1 overlay experiments are shown in **Figure 10B**. The band pattern of mucus components recognized by Msgal1-L1 was variable depending on the health status of individual fish sampled, specifically if they were either apparently uninfected and healthy or severely infected with *M. marinum*. In the uninfected healthy animals (**Figure 10B**, Lane A), rMsgal1-L1 strongly recognized a cluster of mucus components between 55 and 75 kDa, and less intensely, components between 37 and 45 kDa. In infected animals (**Figure 10B**, Lane B), rMsgal1-L1 strongly recognized a 26-kDa component, and weakly bound to mucus components clustering approximately 55–75 kDa and 37–45 kDa. The complex band pattern observed with ConA, which recognizes glycans with exposed high mannose structures, with multiple bands within the 45–50 kDa, 60–70 kDa, and higher MW, revealed the ample diversity of glycosylated components in striped bass epidermal mucus. In summary, rMsgal1-L1 only bound to selected components of striped bass mucus, with variable band patterns showing differences in mobility and intensity between uninfected healthy and infected animals.

### Msgal1-L1 selectively binds to environmental bacteria

To investigate the possibility that in addition to binding to the mucus carbohydrate matrix, Msgal1-L1 would also recognize and bind to environmental bacteria, the binding of Msgal1-L1 to selected bacterial species was first examined by agglutination. Subsequently, the binding of rMsgal1-L1 to bacterial glycans such as LPS and exopolysaccharides was investigated by MGM. Finally, based on the results of the MGM analysis, the relative binding of rMsgal1-L1 to whole cells from three selected bacterial species (*E. coli*, *S. pneumoniae*, and *M. marinum*) was examined by ELISA.

#### Bacterial agglutination by rMsgal1-L1

For the agglutination assays, both Gram-positive and Gram-negative bacteria, that included species known as pathogens for striped bass, were selected and tested for binding by rMsgal1-L1 (1 mg/mL in PBS), comparing the agglutination patterns observed with controls consisting in either bacterial suspensions with added buffer alone or with rMsgal1-L1 (1 mg/mL in PBS) that had been preincubated with lactose (200 mM). Bacteria that were strongly agglutinated by Msgal1-L1 under the assay conditions were *C. piscicola*, *Edwardsiella* sp., *P. shigelloides*, *S. faecalis*, *V. anguillarum*, *V. cholerae*, *V. mimicus*, *V. parahaemolyticus*, and *V. vulnificus* (**Table 3**). Five bacterial species tested (*P. damselae, S. aureus*, and *A. hydrophila*) spontaneously aggregated in small clumps in the controls lacking added rMsgal1-L1, making their results inconclusive. Bacteria that were not agglutinated by rMsgal1-L1 were *A. veronii*, *A. trota*, *B. subtilis*, and *P. aeruginosa*.

**Table 3:** Binding of the purified Msgal1-L1 to bacterial species and strains.

Bacterial species	Agglutination
Aeromonas hydrophila*	+
*A. veronii*	−
*A. trota*	−
*Carnobacterium piscicola*	+
*Edwardsiella sp.*	+
*Vibrio anguillarum*	+
*V. mimicus*	+
V. cholerae*	+
V. parahemolyticus*	+
*V. vulnificus*	+
Photobacterium damselae*	+
*Bacillus subtilis*	−
*Pseudomonas aeruginosa*	−
*Plesiomonas shigelloides*	+
Staphylococcus aureus*	+
*Streptococcus faecalis*	+

Msgal1-L1 binding to bacteria was analyzed by an agglutination microplate assay. Multiple G+ and G− bacterial pathogens and bacterial species considered as components of estuarine environmental consortia, some recognized pathogens of fish, were strongly agglutinated by Msgal1-L1, while several species were not recognized. *Light to moderate bacterial self-agglutination was observed in the negative control (no Msgal1-L1 added).

#### Analysis of rMsgal1-L1 binding to microbial glycans by microbial glycan microarray.

The binding profile of rMsgal1-L1 to purified bacterial components printed in the MGM developed by the NCFG, Harvard Medical School, provided information about the nature of the moieties recognized on the bacteria. The strongest binding observed was to the capsular exopolysaccharide of *S. pneumoniae* type 14 (Danish type 14), while other structures recognized included O-antigen polysaccharide (OPS) from *Proteus* species, and exopolysaccharides from *Shigella flexneri* type 4a and *Proteus mirabilis* O3ab (S1959) (**Table 4**).

**Table 4:** Binding of the purified Msgal1-L1 to bacterial glycans in a microbial glycan microarray.

Chart #	BPS #	Bacteria/Strain	Name/Structure/Cat. No.	Average	SD	%CV
234	243	*Streptococcus pneumoniae* type 14 (Danish type 14)	197-X//Capsular PS	21,205	1,997	9
100	108	*Proteus mirabilis* O28 (PrK 51/57)	OPS	3,206	279	9
99	107	*Proteus mirabilis* O27 (PrK 50/57)	OPS	2,770	255	9
37	42	*Proteus mirabilis* O3a, 3c (G1)	OPS	2,676	484	18
282	291	*Shigella flexneri* type 4a	PS	2,660	385	14
117	125	*Proteus penneri* O67 (8)	OPS	1,979	246	12
103	111	*Proteus mirabilis* O41 (PrK 67/57)	OPS	1,917	290	15
261	270	*Proteus mirabilis* O3ab (S1959)	PS	1,713	558	33
95	102	*Proteus mirabilis* O16 (4652)	OPS	1,548	190	12
96	103	*Proteus mirabilis* O17 (PrK 32/57)	OPS	1,334	706	53
98	106	*Proteus mirabilis* O26 (PrK 49/57)	OPS	1,102	106	10
120	128	*Proteus penneri* O73a,b (103)	OPS	802	13	2

Msgal1-L1 binding to bacterial glycans was analyzed by a microbial glycan microarray. The galectin was selective for glycans from bacterial species considered as components of estuarine environmental consortia, some recognized pathogens of fish.

#### Analysis of rMsgal1-L1 binding to selected bacterial species

**Figure 11:**
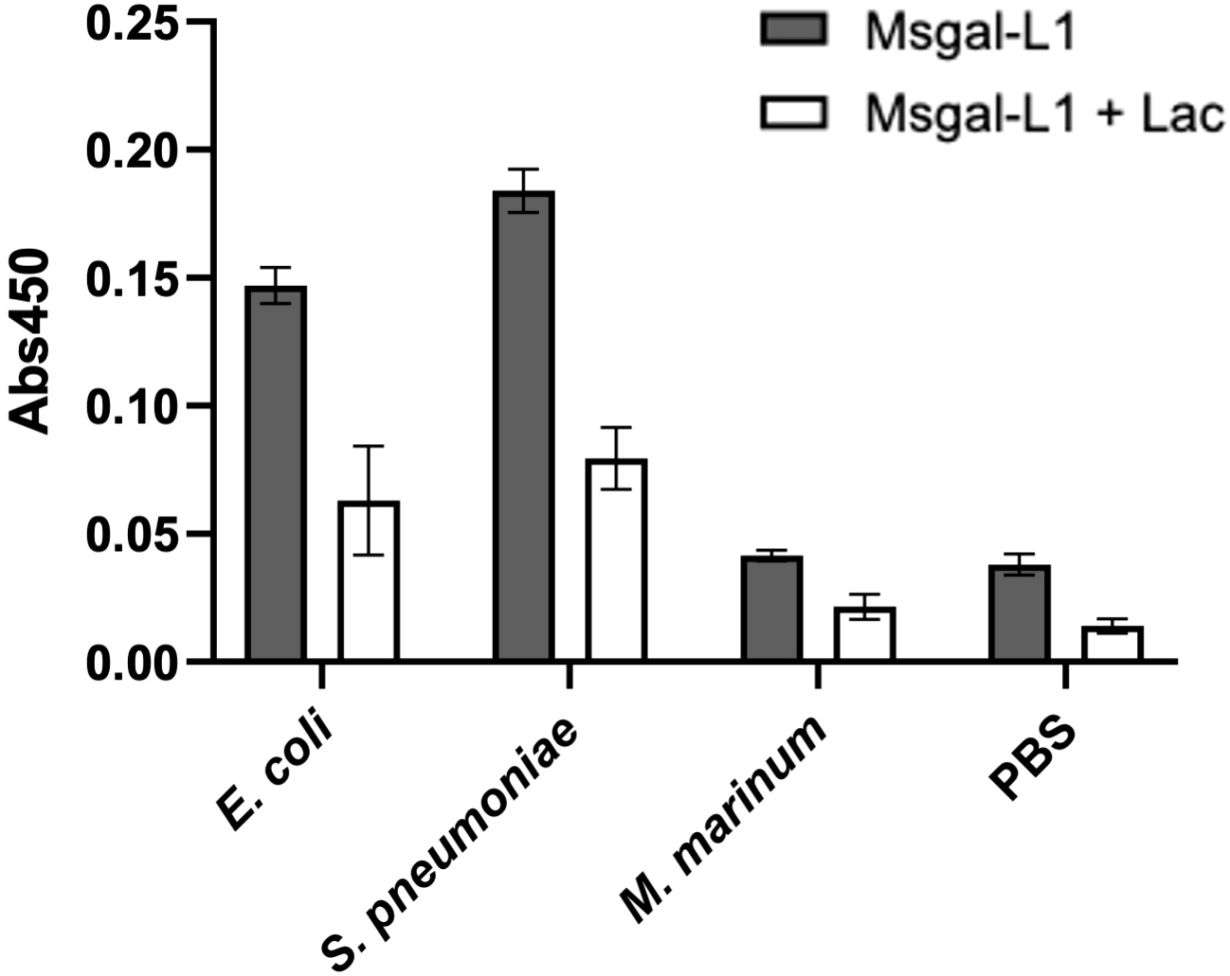
Binding of Msgal1-L1 to bacteria in a solid-phase assay. *E. coli*, *S. pneumoniae*, and *M. marinum* were immobilized in EIA plates as described in Experimental Procedures, washed twice with PBS and PBS-T, and incubated with biotinylated rMsgal1-L1 (with or without lactose) or with PBS alone (with or without lactose) for 1 h at RT. The wells were washed thoroughly and incubated with streptavidin-HRP, developed with HRP substrate, and the absorbance was measured at 450 nm. Absorbance values of the background [no rMsgal1-L1 (only PBS) with or without lactose] were averaged and subtracted from the absorbance values for rMsgal1-L1 (with or without lactose) for each bacteria species. The figure shows representative results from two independent experiments. Bars represent binding signals from duplicate wells analyzed on GraphPad Prism software (v. 9.5.1).

A preliminary study of the potential binding of Msgal1-L1 to whole cells from selected bacterial species (*E. coli*, *S. pneumoniae*, and *M. marinum*) was carried out by ELISA, with the bacteria immobilized on the wells of 96-well ELISA plates. These bacterial species were selected based on either their well-characterized interactions with galectins (*E. coli*), the positive recognition of their glycosylated products in the MGM (*S. pneumoniae*), or their well-established pathogenicity for striped bass (*M. marinum*). The results revealed that rMsgal1-L1 binds strongly to *E. coli* and *S. pneumoniae*, but binding to *M. marinum* was negligible and similar to the PBS control (**Figure 11**).

## Discussion

In this study, we purified and characterized a 15-kDa galectin (Msgal1-L1, *M. saxatilis* galectin1-Like protein 1) from muscle, skin, and epidermal mucus of striped bass (*M. saxatilis*). The high percent identity/similarity of the translated nucleotide sequence of the complete coding region for Msgal1-L1 with galectins from teleost fish (e.g., bastard flounder 74%/86% and pufferfish 73%/82%) supports the inclusion of Msgal1-L1 in the proto type galectin group reported for teleost species, including the electric eel *E. electricus* (Levi and Teichberg, 1981; Paroutaud et al., 1987), Japanese eel *Anguilla japonica* (Tasumi et al., 2004), conger eel *Conger myriaster* (Ogawa et al., 1999; Muramoto and Kamiya, 1992), bastard flounder *P. olivaceus* (Liu et al., 2013), zebrafish *D. rerio* (Ahmed et al., 2004), the cichlid *Haplochromis* sp (Watanabe et al., 2004), and others available in databases of various genome sequencing projects, such as catfish *Ictalurus punctatus*, medaka *Oryzias latipes*, and rainbow trout *Oncorrhynchus mykiss* (The Institute for Genome Research, www.tigr.org); the Japanese pufferfish *Takifugu rubripes* and the green spotted pufferfish *Tetraodon nigroviridis* (International Fugu Genome Consortium, www.fugu-sg.org/project/info; Center for Genome Research, genome.jgi-psf.org/fugu); and the stickleback *Gasterosteus aculeatus* (Stanford Genome Evolution Center). The Msgal1-L1 sequence also revealed the lack of a leader sequence typical for other described galectins and further supports the notion of the structural conservation of proto type galectins along vertebrate lineages. Moreover, the gene organization of Msgal1-L1 was consistent with proto type galectins from other vertebrates, from teleost fish to mammalian Gal-1, including the chicken galectin-14 (Ohyama and Kasai, 1988), mouse Gal-1 (Chiariotti et al., 1991), and human Gal-1 (Gitt and Barondes, 1991), only differing in the intron sizes. Analysis of the 1,800-bp upstream non-coding region of *Msgal1-L1* revealed that both the 5′-upstream region and intron I possesses numerous potential transcription factor binding sites, including modules such as NFAT/Ap1, present in immune effector cells and involved in upregulating cytokine expression. Importantly, the phylogenetic analysis of Msgal1-L1 revealed that it clustered with proto type galectins of other vertebrates, including teleost fish, amphibian, bird, and mammalian species. Clustering of teleost species was mostly in agreement with the current taxonomy at the level of order. Species within the order Anguilliformes grouped together, as did those in the superorder Percomorpha and order Salmoniformes. Exceptions were galectins from medaka and stickleback (Percomorpha), and the galectin Drgal1-L3 from zebrafish (Cypriniformes). Because of the genomic duplication events in the teleost lineage (Glasauer and Neuhauss, 2014), however, most species express multiple proto type galectins, and it is possible that sequences included in the analysis are products of extant paralogous (rather than orthologous) galectin genes.

The structural model of Msgal1-L1 was based on the zebrafish galectin DrGal1-L2 structure as a template (Ghosh et al., 2019). The sequences of Msgal1-L1 and Drgal1-L2 were 60.61% identical and showed no differences in the carbohydrate binding site, with all residues that interact with the carbohydrate ligand fully conserved. The substitution of a histidine by cysteine 53 in the Msgal1-L1 CRD, however, may not only contribute to the susceptibility to oxidative inactivation of Msgal1-L1 but also to the difference in overall distribution of surface positive and negative charges observed between Drgal1-L2 and Msgal1-L1, which may influence the binding of Msgal1-L1 to viral and bacterial glycoproteins, and hinder their attachment to the host epithelial cell surface, which is vital for infection.

The structural similarities in the CRD between Msgal1-L1 and Drgal-L2 were reflected in their similar carbohydrate specificity. Furthermore, a comparison of the binding inhibition by several mono- and oligosaccharides of the two teleost fish prototype galectins, Msgal1-L1 and Drgal-L2, with proto type mammalian (bovine, *B. taurus*) and amphibian (toad, *B. arenarum*) galectins revealed a similar basic pattern of specificity relative to lactose, with slight to moderate variation in the actual inhibitory concentrations. The relative inhibitory efficiencies of four key oligosaccharide structures in the order *N*-acetyllactosamine (Gal1,4GlcNAc) > lactose> human blood group A-tetrasaccharide > Gal1,3GalNAc have been suggested as a way to group galectins as “Type I” (conserved) or “Type II” (variable) in reference to the primary structure of the CRD (Ahmed and Vasta, 1994). The effectiveness of *N*- acetyllactosamine was threefold higher than lactose, with A-trisaccharide threefold less effective than lactose, and the effect of Gal1,3GalNAc negligible. Thus, Msgal1-L1 can be grouped with the galectins that have the “Type I” CRD. The microarray analysis confirmed that Msgal1-L1 prefers binding to non-reducing terminal *N*-acetyllactosamine units in an oligosaccharide, but it can also recognize internal *N*-acetyllactosamine units. The solid-phase binding-inhibition experiments revealed that a hydrophobic phenyl group linked to the reducing end of Galβ1,3GlcNAc substantially strengthened the Msgal1-L1 interaction with the ligand, suggesting additional hydrophobic interactions of the ligand with the protein. Besides the canonical residues of the CRD, it has been proposed that an extended binding site allows for greater affinity for oligosaccharides longer than the disaccharide Gal1,4Glc or Gal1,4GlcNAc (Liao et al., 1994), most notable in Gal-3 (Seetharaman et al., 1998). The sequence of Msgal1-L1 suggests the presence of an extended binding site but displays a fine carbohydrate specificity more similar to the mammalian galectin-1 (Ahmed et al., 1996; Liao et al., 1994) than to Gal-3 (Seetharaman et al., 1998). Of the posited ligands for prototype galectins, the two major ligands appear to be poly-*N*-acetyllactosamine-enriched glycoconjugates found ubiquitously on the cell surface, and poly-*N*- acetyllactosamine extensions on O-glycans from mucins (Wilkins et al., 1996).

The thermal stability analysis of Msgal1-L1 revealed that the activity of Msgal1-L1 does not decrease in the range of temperatures that striped bass thrive. Striped bass eggs and larvae cannot survive outside the range of 10°C and 26°C, and juveniles experience zero growth outside the range of 10°C and 33°C (Cox and Coutant, 1981; Coutant, 1985). There is no reported lethal temperature for striped bass, but there is a downward shift in optimal temperature as the fish age (Coutant, 1985). Within these temperature ranges, Msgal1-L1 retains almost all its activity, and the gradual decline in binding activity observed at higher temperatures is most likely due to unfolding and denaturation of the globular CRD. The thermostability profile of the toad (*B. arenarum*) galectin (Ahmed et al., 1996) has a similar pattern, but the activity of toad galectin does not reach zero till ~84°C. The thermostability of the congerins (I and II) is much greater, remaining fully active until 50°C and 45°C, respectively, and having total loss of activity at 70°C and 60°C, respectively (Muramoto and Kamiya, 1992; Muramoto et al., 1999; Nakamura et al., 2001). The binding activity of Msgal1-L1 was optimal within the pH 7.75–8.25 range, which would correspond to the physiological pH of the internal milieu. The lower binding activity of Msgal1-L1 outside of the above mentioned pH range would be due to the disruption of salt bridges and electrostatic interactions established between the protein and the sugar ligand, as well as hydrophobic interactions responsible for subunit dimerization, as identified in the crystal structure of the mammalian galectin (Liao et al., 1994). The lack of activity at the extremes of the pH range tested would be due to added protein denaturation (Ahmed et al., 1996). Msgal1-L1 was gradually inactivated by exposure to an oxidative environment, likely caused by oxidation of the free cysteines and/or tryptophan in the binding site. Mammalian galectins, which possess six conserved cysteines, are very unstable in the oxidative extracellular environment (Ahmed et al., 1996). The zebrafish galectin Drgal1-L2 possesses three cysteines (Ahmed et al., 2004), and congerin I and II (Muramoto and Kamiya, 1992; Muramoto et al., 1999; Nakamura et al., 2001) and electrolectin, a galectin from the electric eel *E. electricus* (Levi and Teichberg, 1981), possess no cysteines, which makes them less susceptible to oxidative inactivation.

Immunohistochemical analysis revealed that Msgal1-L1 is ubiquitously expressed throughout tissues of adult striped bass, including skin, gills, muscle, and all sampled regions of gut, such as esophagus, pyloric caeca, stomach, and intestine. Not only did macrophage- and fibroblast-like cells stain strongly with the anti-Msgal1-L1 antibody, but also did the surrounding connective tissue, suggesting that it displays glycosylated ligand(s) for secreted extracellular Msgal1-L1. Based on both the carbohydrate specificity of Msgal1-L1, and the ligands reported for proto type galectins from other species, the most likely ligands are laminin and cell-surface α/βintegrins (Gu et al., 1994; Akimoto et al., 1995; Heilmann et al., 2000; Uehara et al., 2001; Ahmed et al., 2004).

Most notably, however, active Msgal1-L1 was abundant in epidermal mucus and could be readily purified from the soluble mucus fractions. Although galectins have been isolated from the mucus of several fish species, the most detailed studies have been carried out for the conger eel (Muramoto and Kamiya, 1992; Muramoto et al., 1999; Nakamura et al., 2001), in which two galectins (congerin I and congerin II) were localized to the skin and mucus (Muramoto and Kamiya, 1992; Muramoto et al., 1999; Nakamura et al., 2001), and the Japanese eel, in which the galectin AJL-1 was expressed in skin mucus-producing cells and secreted into mucus (Tasumi et al., 2004). The expression and release of galectins by mucus-producing cells have also been reported for human intestinal epithelial goblet cells (Gaudier et al., 2004). The striped bass goblet cells that we examined, however, showed no staining for Msgal1-L1, suggesting that although these are mucus-producing cells, they may not be the source of the Msgal1-L1 present in epidermal mucus.

Thus, the question remained open as to the source of soluble Msgal1-L1 we purified from epidermal mucus. In this regard, we speculated that Msgal1-L1 may be actively secreted from epithelial cells of the epidermis, passively released from dead epithelial cells that have been sloughed off into the mucus, or from active macrophages present on the epithelial surface and mucus. In our immunohistochemical analysis of skin and gill sections, both the epithelial cells and cells morphologically similar to tissue-resident macrophages or fibroblasts were intensely stained for Msgal1-L1. When probed with the anti-Msgal1-L1 antibody, the mucus matrix was stained lightly, while the sloughed, and most likely lysed, epithelial cells present in the epidermal mucus did not stain at all or very weakly at most, as compared to the strongly stained intact macrophage-like cells. This suggested the possibility that in addition to the epithelial cells, these macrophages are an additional source of the soluble Msgal1-L1 present in mucus. In this regard, the presence of abundant Msgal1-L1 in the cytoplasm of macrophages isolated from the striped bass head kidney strongly supports this possibility. The expression of galectins, including Gal-1, in epithelial cells and their release to the extracellular environment have been documented in multiple reports (Bao and Hughes, 1995; Bürger et al., 1996; Oka et al., 1999; Nio et al., 2005; Ahmed et al., 2007; Iqbal et al., 2024). Despite lacking a signal sequence, galectins are secreted to the extracellular space by an unconventional, not fully understood mechanism that does not involve the classical secretory pathway, and can bind to the cell surface glycocalyx in an autocrine-like manner (Bänfer et al., 2022; Cummings et al., 2022). Similarly, several publications have described the expression of galectins in macrophages and their secretion to the extracellular space, where they would be involved in regulatory functions related to both innate and adaptive immunity (Rabinovich et al., 1999; Dias-Baruffi et al., 2003; Dhirapong et al., 2008). In mammals, Gal-1 is expressed by activated macrophages and secreted to the extracellular space where it functions in promoting phagocytosis of neutrophils by activated macrophages (Dias-Baruffi et al., 2003) and in signaling T-cell apoptosis (Rabinovich et al., 1999). Gal-1 has also been localized to the cytoplasm and nucleus of dermal fibroblasts (Akimoto et al., 1995). Furthermore, it has been reported that in addition to regulatory functions, galectins secreted to the extracellular environment also carry out direct recognition of potential pathogens and parasites (Vasta, 2009; Vasta et al., 2012, 2017).

Msgal1-L1 recognized epidermal mucus glycans in a carbohydrate-specific manner susceptible to inhibition by lactose. Human Gal-1 and Gal-3 bind to *N*-acetyllactosamine moieties on N-linked and O-linked oligosaccharides of both soluble and cell-associated mucins (Bresalier et al., 1996; Seelenmeyer et al., 2003; Gadi et al., 2025; Cummings et al., 2022). As shown by the dot-blot experiments with glycosidase-treated mucus, Msgal1-L1 recognized oligosaccharides from mucus-soluble mucins that are susceptible to cleavage by glycosidases. While N-glycosidase F releases N-linked oligosaccharide structures, O-glycosidase releases the disaccharide Galβ(1–3)GalNAc from O-glycans attached to serine or threonine, and Endo H shows considerable specificity for N-linked structures such as high mannose and hybrid-type oligosaccharides. As Msgal1-L1 strongly bound to the untreated viscous fraction of striped bass mucus, deglycosylation by N-glycosidase F only moderately decreased binding, but treatment with O-glycosidase and endoglycosidase H drastically reduced Msgal1-L1 binding. This suggested that Msgal1-L1 binding to mucus was mostly via O-linked oligosaccharides. The overlay experiments revealed that the oligosaccharides recognized by Msgal1-L1 are associated to a rather restricted group of mucus components, most likely mucins rich in O-linked sugars.

As revealed by agglutination assays, Msgal1-L1 can directly recognize and agglutinate various environmental Gram-positive and Gram-negative bacterial species and strains in a carbohydrate-dependent manner, some of which are well-known fish pathogens. Based on the microbial microarray analysis, interactions of Msgal1-L1 with bacteria would take place by recognition and binding to capsular exopolysaccharides and lipopolysaccharide O-antigen oligosaccharides that display galactose moieties accessible to galectin binding. It is noteworthy that a preliminary study showed that Msgal1-L1 strongly bound *Streptococcus* sp., but very weakly to *M. marinum*, an endemic pathogen of striped bass in Chesapeake Bay.

Taken together, the evidence obtained from our study suggests that Msgal1-L1 may function in innate immune defense against potentially pathogenic bacteria by agglutinating and/or cross-linking them to mucins in the epidermal mucus and immobilizing them within the mucus film matrix to prevent their access to the fish epithelial cell surface and eliminate them with the periodically sloughed off mucus. It is also possible that Msgal1-L1 present in mucus acts as an opsonin for potential pathogens to be phagocytosed by mucosal macrophages in a similar manner as the lung collectins in the airway mucus opsonize inhaled pathogens to alveolar macrophages (Patel et al., 2024; Smole et al., 2020) or the extracellular galectins opsonize *Perkinsus* spp. parasites to the oyster mucosal phagocytic hemocytes (Harvell et al., 1999; Feng et al., 2013; Shi et al., 2014; Feng et al., 2015; Vasta and Wang, 2020). Thus, consistent with a “Red Queen” effect, it is tempting to speculate that *Mycobacterium* spp. may have co-evolved with the fish host to evade this defense mechanism, to reach and infect the fish skin epithelial layer leading to ulcers and granulomas frequently observed in the epizootic mycobacteriosis in both farmed and wild striped bass populations.

## Data Availability

The gene and transcript have been deposited in NCBI. The accession numbers are BankIt2947350 BSeq#1 PV533650 and BankIt2947352 BSeq#1 PV533651.

## Supplementary material

Supplementary Figure 1 Enterokinase treatment of rMsgal1-L1: rMsgal1-L1 was purified from supernatant of lysed bacterial culture (IPTG-induced) and treated with enterokinase as described in Experimental Procedures. Cleavage of the rMsgal1-L1 was monitored at various time points by SDS-PAGE, until no 19-kDa protein was detected. The resin with the bound protein was washed to remove enzyme and unbound cleavage products, and eluted with PBS/lactose. Lane 1: Uninduced bacterial lysate; Lane 2: IPTG-induced bacterial lysate; Lane 3: Lactose eluate of the bound protein:lactosyl-Sepharose resin (no enterokinase added); Lane 4: Lactose eluate of the bound protein:lactosyl-Sepharose resin (incomplete enterokinase cleavage); Lane 5: Lactose eluate of the bound protein:lactosyl-Sepharose resin (complete enterokinase cleavage).

Supplementary Figure 2 Immunohistochemical localization of Msgal1-L1 in skin and gill tissues. Sections (4 μm thick) of skin and gills mounted on APES coated slides were either stained with PAS or incubated with anti-Msgal1-L1 or pre-immune serum (as control), as described in Experimental Procedures. Bound antibody (anti-Msgal1-L1 and pre-immune serum control) was visualized using Stable DAB.

Supplementary Figure 3 Immunohistochemical localization of Msgal1-L1 in microvilli of the pyloric caeca. Sections (4 μm thick) of the pyloric caeca were mounted on APES coated slides and processed as described in **Supplementary Figure 2**. Labeled subcellular structures are as follows: M-LC, Macrophage-like cells; G, Goblet cells; LP, Lamina propria.
