# Supplementary material for: A “proto” type galectin expressed in striped bass (Morone saxatilis) tissues is released to epidermal mucus and binds to bacterial and mucus glycans
Source: Front Cell Infect Microbiol. 2025 May 14;15:1572734. doi: 10.3389/fcimb.2025.1572734 (PMC12116657; doi:10.3389/fcimb.2025.1572734)
Supplement: Supplementary Figure 1 — Enterokinase treatment of rMsgal1-L1: rMsgal1-L1 was purified from supernatant of lysed bacterial culture (IPTG-induced) and treated with enterokinase as described in Experimental Procedures. Cleavage of the rMsgal1-L1 was monitored at various time points by SDS-PAGE, until no 19-kDa protein was detected. The resin with the bound protein was washed to remove enzyme and unbound cleavage products, and eluted with PBS/lactose. Lane 1: Uninduced bacterial lysate; Lane 2: IPTG-induced bacterial lysate; Lane 3: Lactose eluate of the bound protein:lactosyl-Sepharose resin (no enterokinase added); Lane 4: Lactose eluate of the bound protein:lactosyl-Sepharose resin (incomplete enterokinase cleavage); Lane 5: Lactose eluate of the bound protein:lactosyl-Sepharose resin (complete enterokinase cleavage). [file DataSheet1.pdf]

Supplemental Figure 1

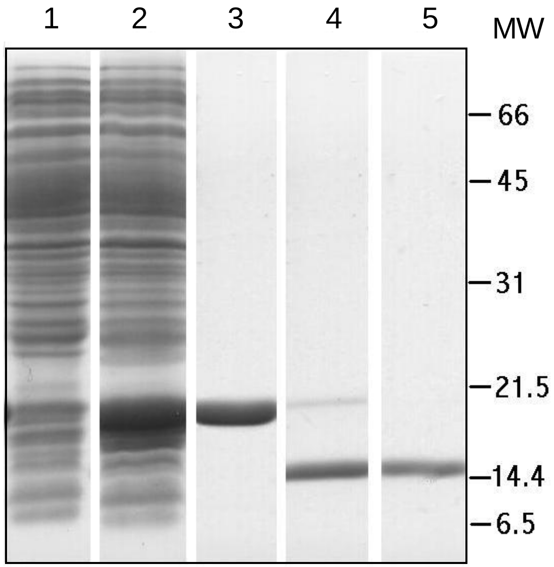

Supplemental Figure 2

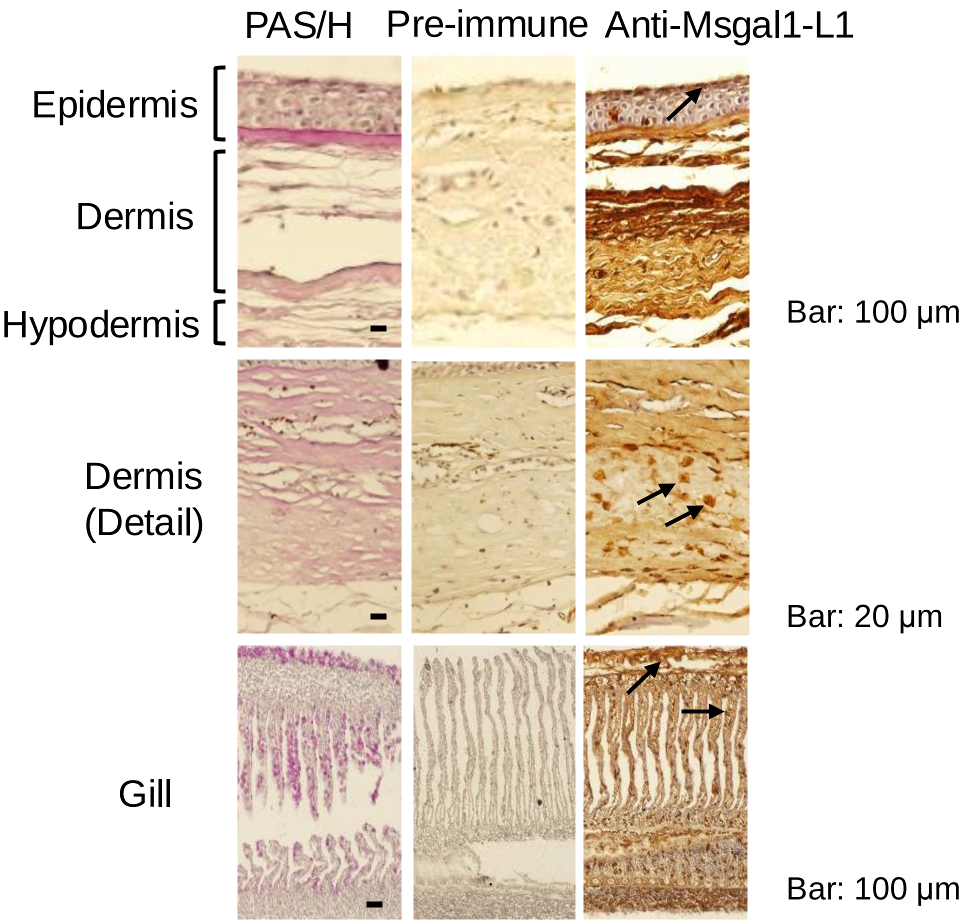

Supplemental Figure 3

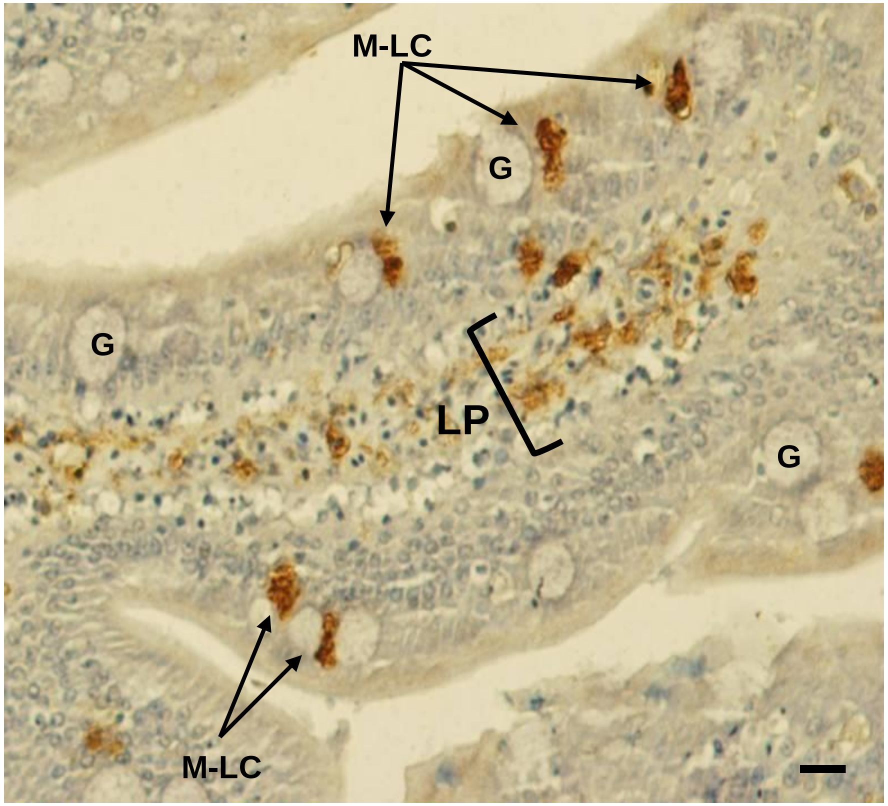

M-LC: Macrophage-like cells
G: Goblet cells
LP: Lamina propria
